# Pentadentate and Hexadentate Pyridinophane Ligands Support Reversible Cu(II)/Cu(I) Redox Couples

**DOI:** 10.3390/inorganics11110446

**Published:** 2023-11-20

**Authors:** Glenn Blade, Andrew J. Wessel, Karna Terpstra, Liviu M. Mirica

**Affiliations:** 1 Department of Chemistry, University of Illinois Urbana-Champaign, 600 S. Matthews Ave, Urbana, IL 61801, USA; 2 Department of Chemistry, Washington University, One Brookings Drive, St. Louis, MO 63130, USA

**Keywords:** bioinorganic chemistry, pyridinophane ligands, copper(II) complexes, copper(I) complexes, cyclic voltammetry, radiolabeling, ^64^Cu PET imaging agents, reversibility

## Abstract

Two new ligands were synthesized with the goal of copper stabilization, N,N′-(2-methylpyridine)-2,11-diaza[3,3](2,6)pyridinophane (^Pic^N4) and N-(methyl),N′-(2-methylpyridine)-2,11-diaza[3,3](2,6)pyridinophane (^PicMe^N4), by selective functionalization of ^H^N4 and ^TsH^N4. These two ligands, when reacted with various copper salts, generated both Cu(II) and Cu(I) complexes. These ligands and Cu complexes were characterized by various methods, such as NMR, UV-Vis, MS, and EA. Each compound was also examined electrochemically, and each revealed reversible Cu(II)/Cu(I) redox couples. Additionally, stability constants were determined via spectrophotometric titrations, and radiolabeling and cytotoxicity experiments were performed to assess the chelators relevance to their potential use in vivo as ^64^Cu PET imaging agents.

## 1. Introduction

Mononuclear copper complexes have been extensively utilized throughout various areas of inorganic chemistry: synthesis of structural or functional biomimetic inorganic complexes of Cu-containing enzymes [1–4], cation detection or sequestration [5,6], development of metal-based therapeutic or diagnostic compounds [7–10], and many others. In particular, the development of ^64^Cu-based positron emission tomography (PET) agents has garnered significant attention in recent years as an alternative to shorter-lived radionuclides ^11^C and ^18^F, which are commonly used in PET imaging [11–13]. However, these ^64^Cu PET agents still face challenges presented by the possibility of in vivo decomplexation. Ideally, a suitable chelator should demonstrate high thermodynamic stability and kinetic inertness in order to avoid this problem, which has been the focus of many studies in recent years [14]. However, a problem faced by some of the most common ^64^Cu chelators is the issue of reduction-induced demetallation. Given the reducing environment of cells and the presence of in vivo bioreductants, the ideal chelator should be able to avoid this issue by remaining stable even upon reduction to Cu^I^ [15]. As such, ligands with flexible donor arms that have the ability to stabilize both Cu^II^ and Cu^I^ are good candidates for chelating ^64^Cu [16]. Several studies have been published in recent years focused on the coordination chemistry of ^64^Cu complexes with macrocyclic ligands substituted with pendant arms like 2-pyridylmethyl, picolinate, thiazolyl, and others [17–21].

In that vein, two new pyridinophane ligand systems, inspired by previous macrocyclic polydentate ligands, have been synthesized [22–24]. By substituting non-interacting groups (i.e. Me or tBu) for groups that can interact with the metal center, greater binding modes than usual for ^R^N4 ligands can be achieved with altered characteristics of the resultant complexes. Interacting groups like 2-methylpyridyl, picolyl—“Pic”, could bind directly with the metal center while being easily synthetically attached to the N4 backbone. When both alkyl groups are chosen to be the picolyl fragment, the resultant hexadentate ligand N,N′-(2-methylpyridine)-2,11-diaza[3,3](2,6)pyridinophane, ^Pic^N4, offers the possibility of a distorted octahedral environment around the metal center while simultaneously shielding from inner sphere interactions. An asymmetric version could also be synthesized using previously reported methods to make N-(methyl),N′-(2-methylpyridine)-2,11-diaza[3,3](2,6)pyridinophane, ^PicMe^N4, which could act as a pentadentate ligand and leave one coordination site available for an exogenous ligand. This flexible pentadentate ligand could more easily adapt to geometries other than a distorted octahedral arrangement. When this ligand was bound to copper, both ^Pic^N4 and ^PicMe^N4 were able to stabilize both Cu^II^ and Cu^I^ oxidation states, with each complex being crystallographically characterized. Each of these four complexes was spectroscopically scrutinized by various techniques, including NMR, EPR, ESI-MS, and UV-Vis. Cyclic voltammetry experiments were able to show that the conversion between Cu^II^ and Cu^I^ was remarkably reversible for both systems, as a consequence of the flexible nature of the picolyl arms being able to come off the Cu center. The ^Pic^N4Cu^II/I^ couple was also low at E_1/2_ = −1.1 V vs. Fc/Fc^+^. Calculation of Cu^II^ stability constants using spectrophotometric titrations also revealed the moderate ability of the complexes to stabilize both Cu^II^ and Cu^I^ complexes. Finally, preliminary radiolabeling studies showed that both ^Pic^N4 and ^PicMe^N4 can quickly and efficiently be radiolabeled with ^64^Cu, making these ligands potentially relevant chelators for use in ^64^Cu PET imaging studies.

## 2. Results and Discussion

### 2.1. Synthesis

The ligand synthesis of ^Pic^N4 was a two-step development. The first attempt at the synthesis of ^Pic^N4 involved an S_N_2-based mechanism utilizing 2-chloromethyl pyridine under basic conditions at a roughly 80% yield [25]. Multiple bases were tested for this synthesis; between sodium carbonate, potassium carbonate, and Hünig’s base (diisopropy-lethylamine), Hünig’s base gave the highest yield of 81%, while the carbonates gave smaller yields of around 40%. Additional synthetic attempts using reductive amination have given inconsistent results and a maximum yield of 46%. Full synthetic details and product descriptions can be found in the Supporting Information.

**Scheme 1.**
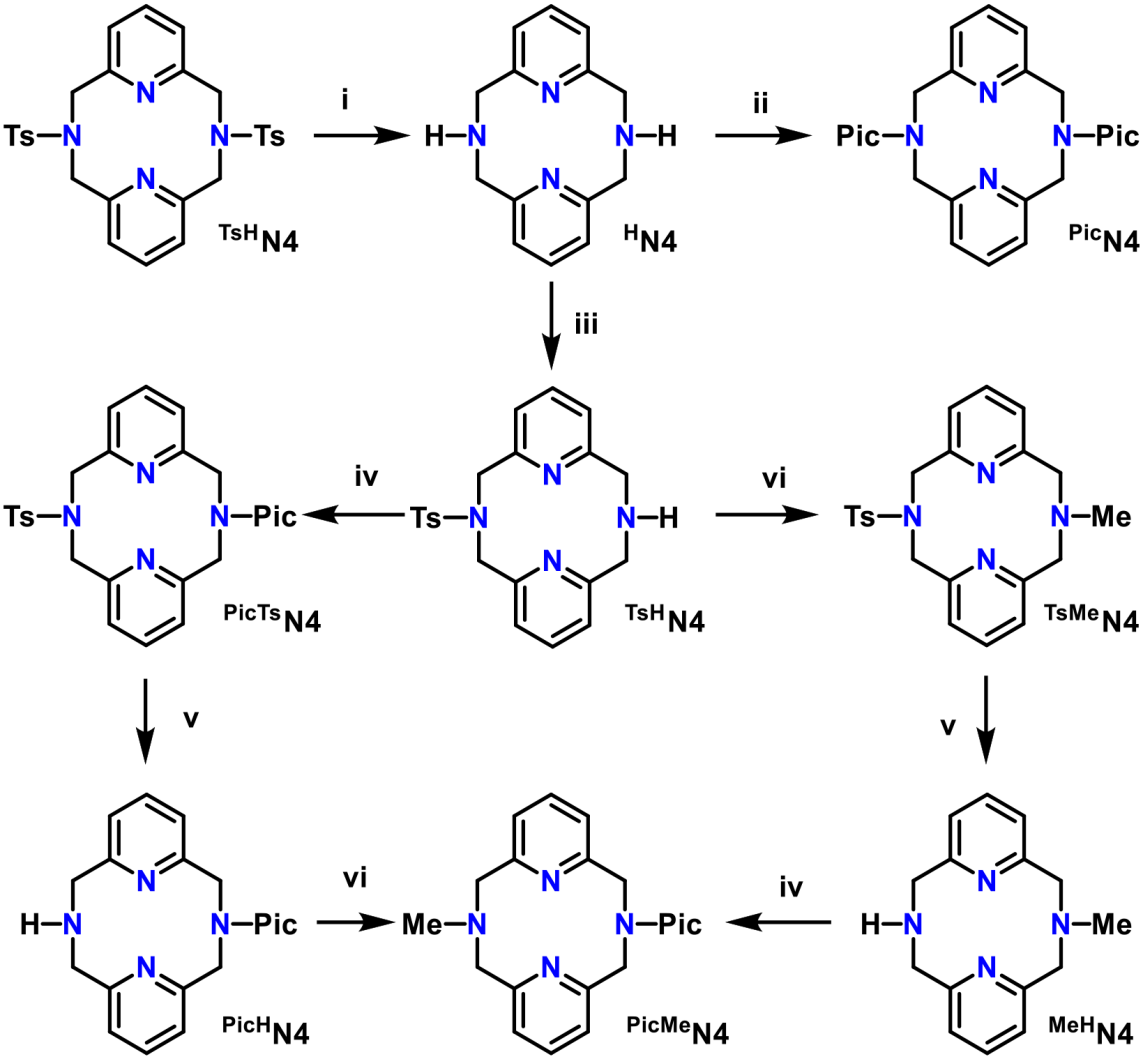
Synthesis of ^Pic^N4 and ^PicMe^N4. (i) 90% H_2_SO_4_, reflux 2.5 h; 88% (ii) 2-(methylchloro)pyridine HCl, iPr_2_EtN, MeCN, 48 h; 81% (iii) TsCl, DCM, 0 C, 3 h; 44% (iv) 2-(methylchloro)pyridine HCl, iPr_2_EtN, MeCN, 48 h; 82% (v) 90% H_2_SO_4_, reflux, overnight; 89% (vi) formic acid, formaldehyde, reflux, overnight; 82%.

Ligand synthesis for ^PicMe^N4 was achieved by two different methods, as depicted in Scheme 1. In the first pathway, the direct functionalization of ^MeH^N4 by placing the 2-methylpyridyl on the secondary amine was performed to make the product. This pathway requires making ^MeH^N4, a product synthesized by previously discussed methods [23]. The second pathway utilized ^TsH^N4 for functionalization to yield ^PicTs^N4. The tosyl deprotection reaction using concentrated sulfuric acid did not degrade the ligand significantly, providing a good yield of ^PicH^N4. The penultimate product, ^PicH^N4, was then methylated to yield the final product, ^PicMe^N4. Since both pathways showed that the products ^TsMe^N4 and ^TsPic^N4 could survive the harsh sulfuric acid conditions of the detosylation reaction, the second pathway was chosen. Functionalization of the secondary amines occurred by one of two methods: reductive amination or S_N_2. Both methods achieved high yields (75% and 81%, respectively), but the S_N_2 was much more consistent and less reliant on the purity of reagents.

**Scheme 2.**
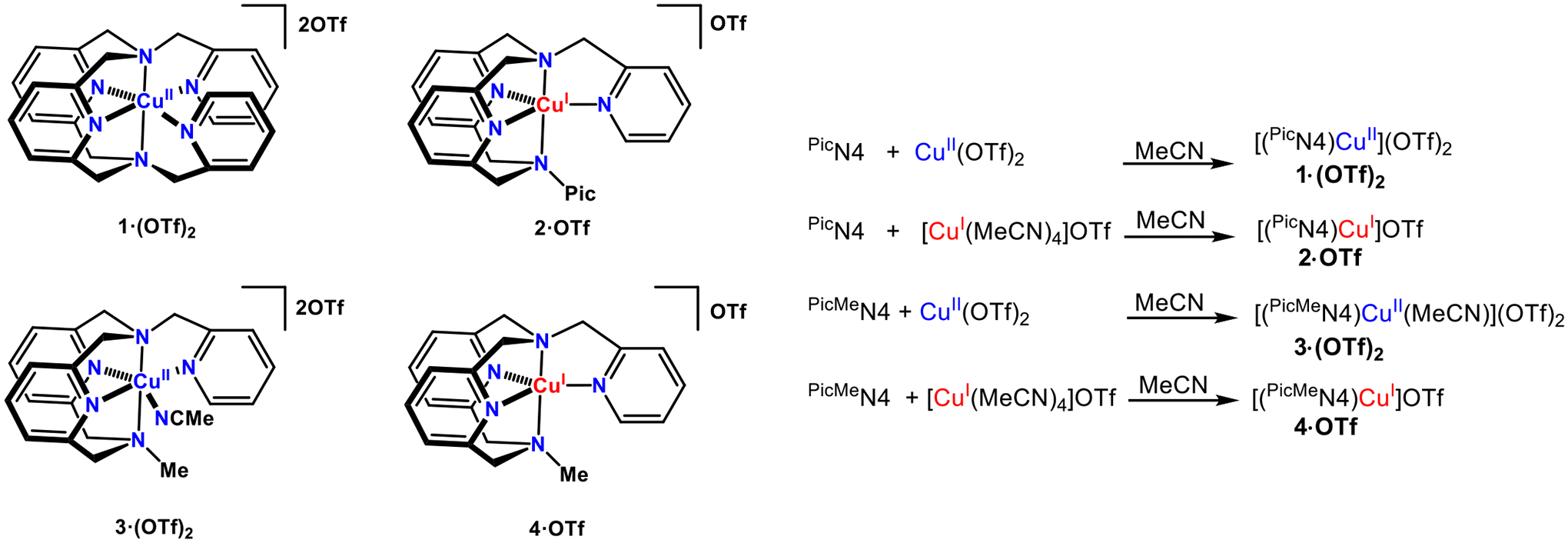
Preparation of Copper Complexes.

The syntheses of the **1·(OTf)**_**2**_ and **2·OTf** complexes was achieved by mixing the appropriate triflate salt with the ligand in MeCN (Scheme 2). Cu^II^(OTf)_2_ and ^Pic^N4 were mixed overnight and either crashed out of solution by trituration with diethyl ether or recrystallized via diethyl ether diffusion, with an 88% yield of **1·(OTf)**_**2**_. While most studies in this paper utilize the triflate complex, other salts like Cu^II^(ClO_4_)_2_ or Cu^II^(PF_6_)_2_ were also employed with similar yields. Similarly, [(MeCN)_4_Cu^I^]OTf and ^Pic^N4 were mixed in MeCN for one hour and recrystallized via ether diffusion for a 55% yield of **2·OTf**.

The synthesis of complexes **3·(OTf)**_**2**_ and **4·OTf** were similarly completed: the relevant copper triflate salt and the ligand were mixed in MeCN (Scheme 2). While crystals for the Cu^II^ salt were not easily obtained, a green solid was crashed out from MeCN with toluene and rinsing with pentane (55% isolated yield). When attempting to get crystals of **3·(OTf)**_**2**_, the use of sodium tetraphenylborate, NaBPh_4_, generated orange crystals of **4·OTf** in low yield. A more acceptable approach to preparing **4·OTf** was ^PicMe^N4 and [(MeCN)_4_Cu^I^]OTf mixed in MeCN for one hour in the dark and recrystallized via diethyl ether diffusion at −35 °C (77% yield).

### 2.2. Structural Characterization of Metal Complexes

**Figure 1.**
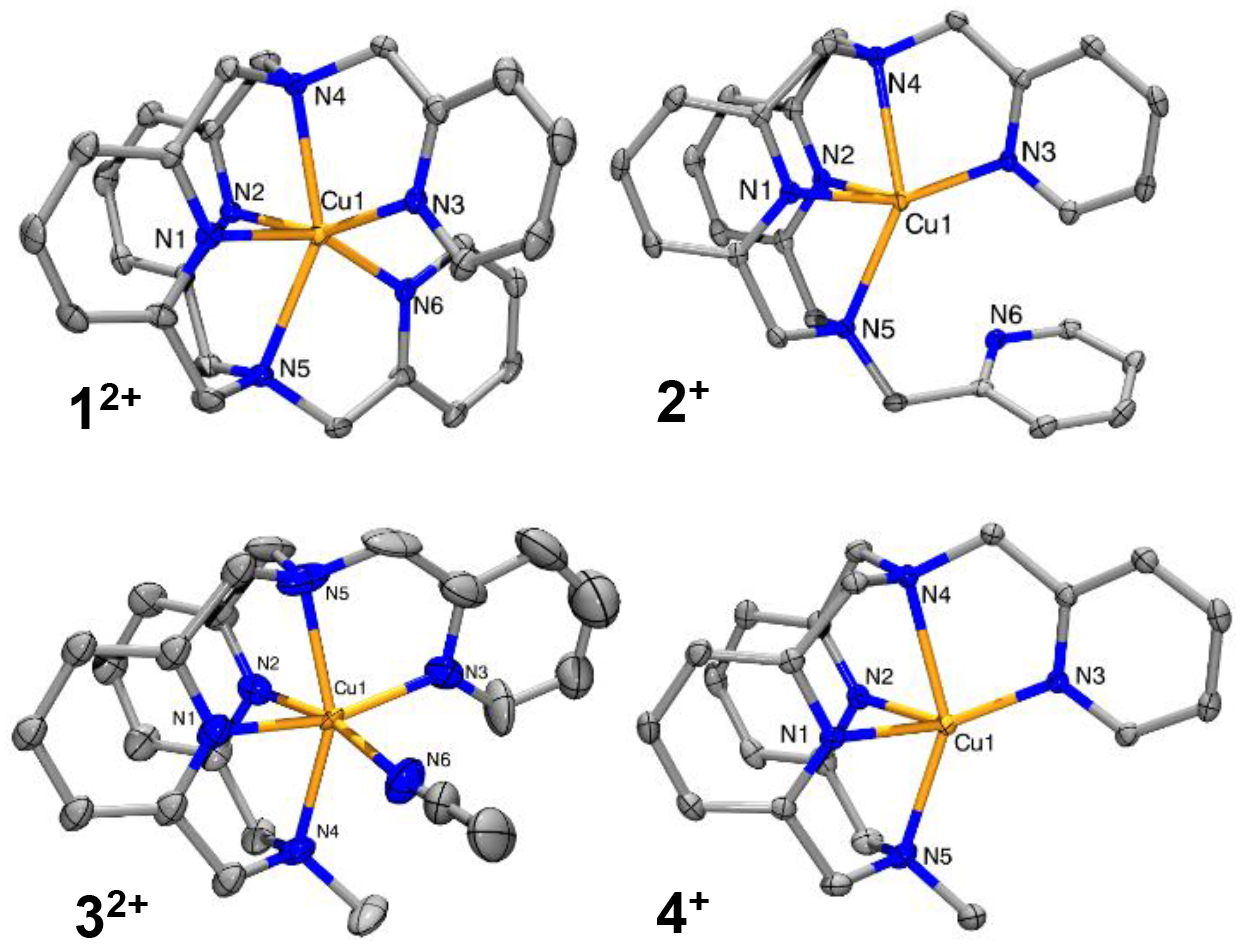
ORTEP plots (50% probability ellipsoids) of cations **1**^**2+**^, **2**^**+**^, **3**^**2+**^, and **4**^**+**^. Counterions and H atoms are omitted for clarity. The crystallographic datasets for **1·(OTf)**_**2**_, **2·OTf**, **3·(OTf)**_**2**_, and **3·OTf** have been deposited at CCDC under the record numbers 2049802, 2049803, 2049804, and 2049805.

X-ray diffraction-quality crystals of the copper complexes were obtained by diethyl ether diffusion into MeCN solutions at room temperature or –35 °C (Figure 1 and Table 1). Full crystallographic details are provided in the Supplemental Information. The crystal structure of **1**^**2+**^ had a similar distorted octahedral environment to the one observed for the analogous ^tBu^N4 complex [22,26,27]. The inclusion of the two pyridine moieties on the metal center preclude the need of additional exogenous ligands, such as solvent or triflates directly bound to the metal center. The Cu^II^ center exhibits a Jahn-Teller like distortion, with the four pyridine nitrogens having relatively short bond lengths to copper (2.00–2.06 Å), while the amine nitrogens form much longer bonds (2.28, 2.35 Å).

Upon reduction to **2**^**+**^, the coordination environment changes to a pentadentate distorted square pyramid geometry with a structural parameter τ_5_ = 0.13.[28] As expected for Cu^I^ structures, the Cu-N_eq_ bond lengths averaged a shorter value of 2.00 Å, while the Cu-N_ax_ bond lengths were much longer at 2.34 Å. The non-coordinating picolyl nitrogen was sufficiently far away to not interact with the Cu center at 3.32 Å [4,22].

Supposing that crystals of **4**^**+**^ was more stable than **3**^**2+**^, the ligand was mixed with Cu^I^OTf in MeCN and recrystallized with ether diffusion at room temperature to yield large orange crystals. The cation of **4**^**+**^ adopts a distorted square pyramid pentadentate geometry with a structural parameter τ_5_ = 0.12, similar to **2**^**+**^ [28]. It has a similar delineation of Cu-N bonds around the Cu: Cu-N_eq_ bond lengths averaged 2.00 Å, while Cu-N_ax_ bond lengths averaged 2.33 Å. Unlike in **2**, the methylamine on the ^PicMe^N4 backbone was less sterically restricted than the picolyl functionalized amines.

In an attempt to obtain crystals of **3·(PF**_**6**_**)**_**2**_, the blue solid was dissolved in MeCN with two equivalents of NaBPh_4_ and subjected to diethyl ether diffusion at room temperature. When isolated, there were primarily orange crystals of **4·PF**_**6**_ present with a blue solution. Crystals of **3·(BPh**_**4**_**)**_**2**_ were eventually recovered under these conditions as a mixture of Cu^II^ and Cu^I^ crystals. Although it was unknown exactly how the Cu^II^ complex was reduced in the solution, it was suspected that the NaBPh_4_ and the BPh_3_ impurity promoted the reduction of the Cu^II^ center. Regardless, **3**^**2+**^ displays a Jahn-Teller like distorted octahedral geometry with one exogenous MeCN bound to the Cu^II^ center. Interestingly, the Cu^II^ species seemed to exhibit Jahn-Teller like compression along the N1-Cu-N3 axis where the Cu-N bond lengths are around 1.95 Å, while the other four Cu-N bonds are longer at an average of 2.20 Å.

### 2.3. Complex Characterization

**Figure 2.**
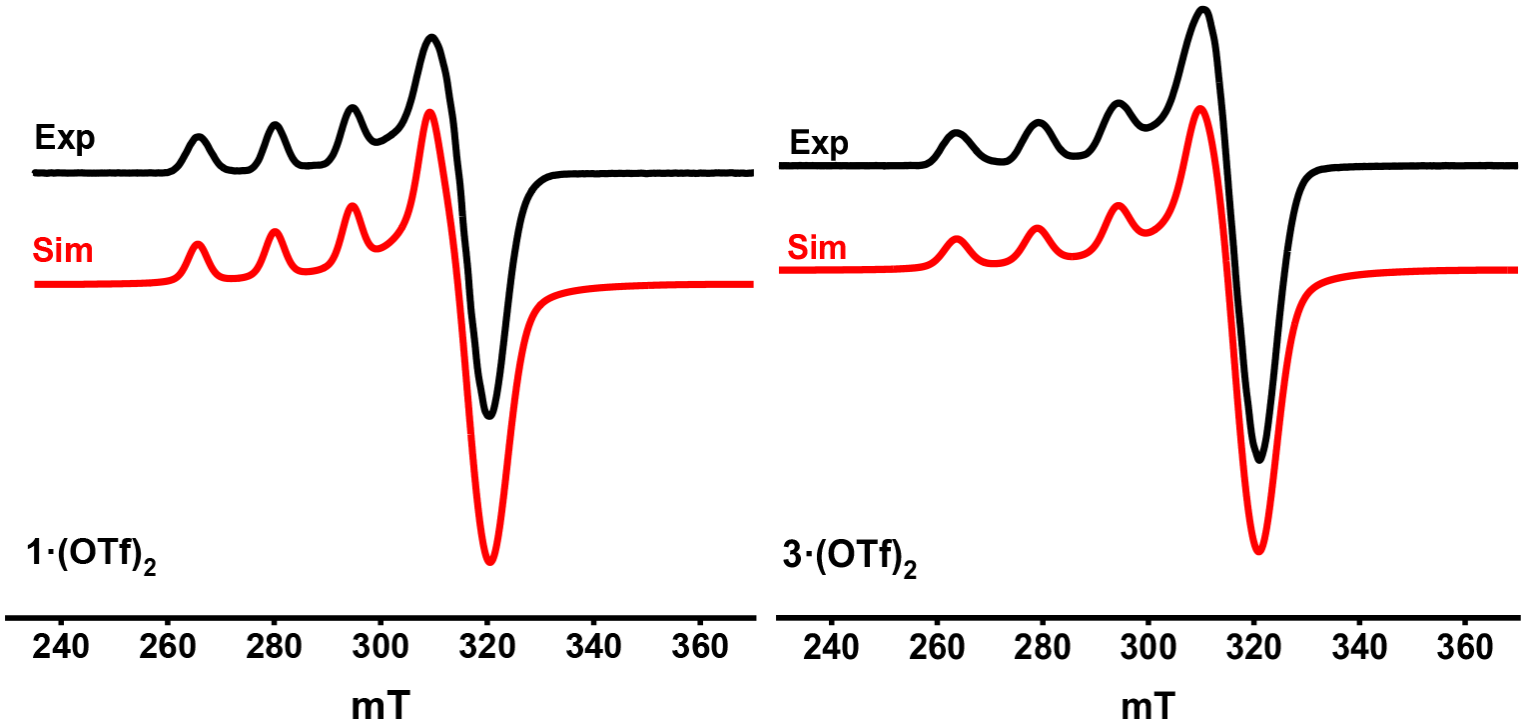
EPR spectrum (black) and simulation (red) of 1·(OTf)_2_ (**left**) and 3·(OTf)_2_ (**right**) in MeCN:PrCN (1:3) at 77K.

To better understand the solution state characteristics of these complexes, several techniques were utilized, including electron paramagnetic resonance (EPR), NMR, and electrochemical studies. The paramagnetic d^9^ Cu^II^ complexes **1**^**2+**^ and **3**^**2+**^ were characterized by EPR in a fashion similar to previous Cu^II^ species, and the EPR spectra are shown in Figure 2 and the EPR parameters are summarized in Table 2. Analysis by Evan’s method measured in CD_3_CN yielded the expected values for these d^9^ Cu^II^ centers: 1.80 μ_B_ and 1.71 μ_B_ for **1**^**2+**^ and **3**^**2+**^, respectively [29]. The EPR spectrum for **1·(OTf)**_**2**_ exhibited values of g_x_ = 2.070, g_y_ = 2.055, and g_z_ = 2.259 (A_z_ = 144.5 G) and g_x_ = 2.067, g_y_ = 2.056, and g_z_ = 2.264 (A_z_ = 152.5 G) for **3·(OTf)**_**2**_, which is consistent with what is expected of a distorted octahedral Cu^II^ center, and in line with the solid state structural data [22,29,30].

While the paramagnetic ^1^H NMR spectrum of **1**^**2+**^ did not afford much information (Figure S14), the spectra of the d^10^ Cu^I^ species could generally be assigned with the help of a gCOSY 2D spectrum (Figures S9–S13). The assignment of the **2·OTf** spectrum gathered in CD_3_CN was assigned as so: the four most downfield aromatic peaks corresponded to the different pyridine hydrogens on the picolyl arm, while the two upfield sets of aromatic multiplets corresponded to the pyridine hydrogens on the N4 backbone [31]. The methylene region contained five total peaks: a singlet (4.62 ppm) matched to the two picolyl methylene hydrogens and a pair of doublets matched to the N4 methylene hydrogens. Further assignment of the N4 methylene hydrogens could not be easily discerned due to the symmetry and structure of the molecule. The integration and the 2D NMR corroborated this assignment (Figure S11). Since the methylene on the picolyl arm appears as a singlet, this implies there was rapid exchange between the bound arm and the unbound arm which was faster than the NMR time-scale.

The assignment of the **4·OTf** spectrum followed in a similar way. The three most downfield aromatic peaks matched the four pyridine hydrogens on the picolyl arm, while the two down-field aromatic multiplets corresponded with the para- and meta-hydrogens on the N4 pyridine backbone. The two singlet peaks in the aliphatic region corresponded to the two methylamines: the methylene moiety on the picolyl arm (4.469 ppm) and the methyl group (3.313 ppm). In a similar fashion to the ^Pic^N4 complex, the methylene on the picolyl arm was not fixed in spaced which allowed resolution into a singlet. The remaining four sets of doublets (J_avg_ ≈ 15 Hz, geminal) corresponded to the methylene protons fixed in place on the N4 backbone. Based on the gCOSY crossover peaks (Figure S13), the doublet pairs 4.248 and 3.668 ppm correspond to interaction protons, while 4.177 and 4.040 ppm are also coupled.

**Figure 3.**
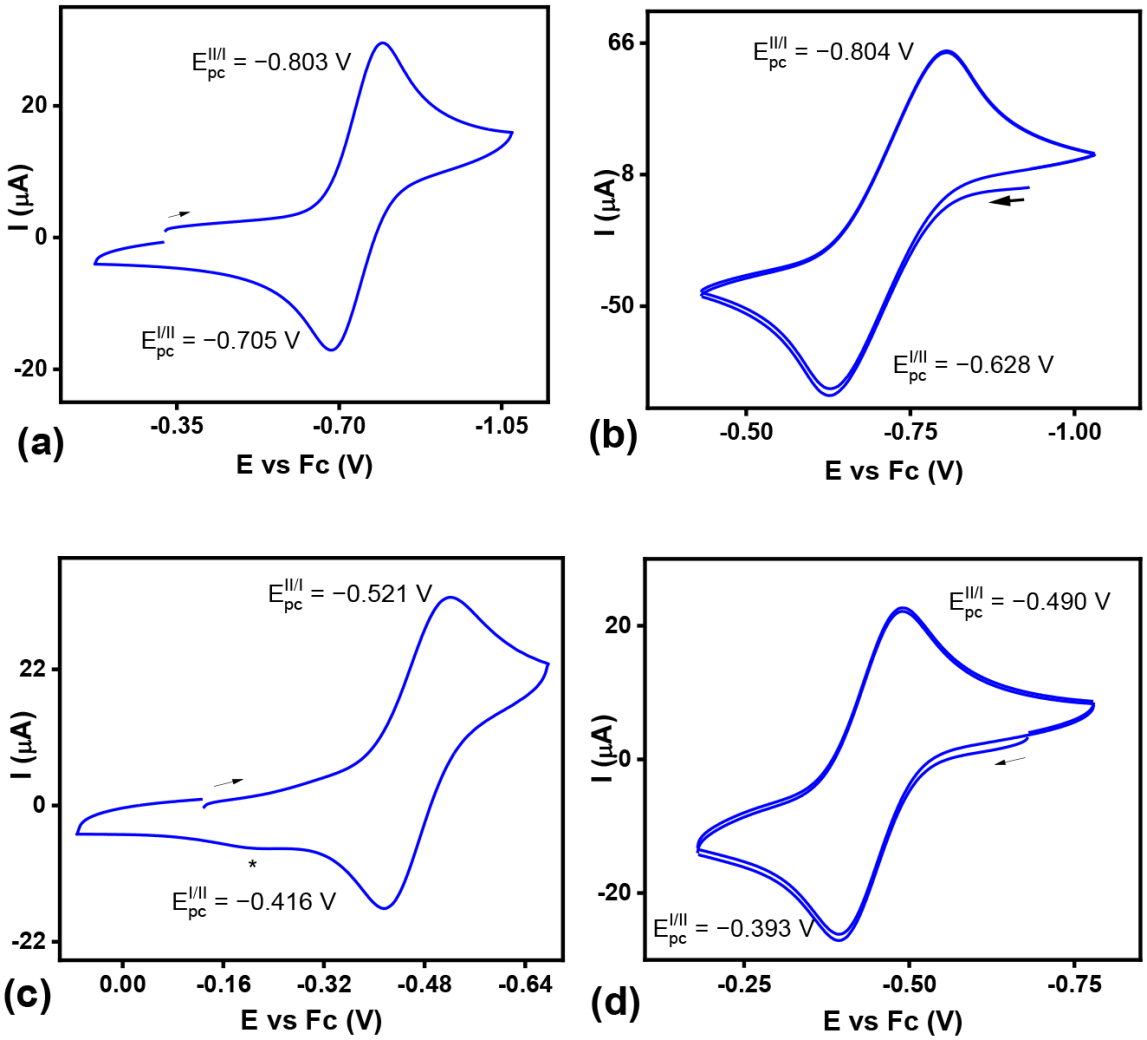
Cyclic voltammetry of the copper complexes **1·(Otf)**_**2**_ (**a**), **2·Otf** (**b**), **3·(Otf)**_**2**_ (**c**), and **4·Otf** (**d**) (0.1 M Bu_4_NclO_4_/CH_3_CN; arrow indicates the initial scan direction). The asterisk (*) corresponds to a trace amount of ^PicMe^N4Cu^II^(H_2_O) complex.

Cyclic voltammetry (CV) for **1·(OTf)**_**2**_ featured a couple at −0.752 V vs. Fc^0/+^ (Figure 3), corresponding to the Cu^II/I^ couple with a quasi-reversible nature (ΔE_p_ = 97 mV) as well as an irreversible oxidation at + 1.047 V (Figure S15). In order to confirm the reversibility of the Cu^II/I^ couple, **2·OTf** was also scrutinized to yield a similar couple at −0.716 V vs. Fc^0/+^ (ΔE_p_ = 176 mV). A similar analysis for **3·(OTf)**_**2**_ found a quasi-reversible Cu^II/I^ couple at −0.468 V vs. Fc^0/+^ (ΔE_p_ = 105 mV) along with an irreversible Cu^III^ oxidation at 1.552 V (Figure S16). Confirming the reversibility of this quasi-reversible Cu^II/I^ couple, a CV of **4·OTf** showed the couple at −0.441 V vs. Fc^0/+^ (ΔE_p_ = 96 mV).

Notably, all four copper complexes exhibit larger ΔE_p_ values than the values expected for fully reversible redox processes (Table 3). However, the measured ΔE_p_ values for the Fc^0/+^ couple in both sets of experimental conditions, 129 mV and 176 mV, are also larger than standard values, indicating that the large peak-to-peak separation may not necessarily imply redox irreversibility of the copper complexes. Furthermore, the discrepancies between the ΔE_p_ values of the complexes can be explained by the different sets of experimental conditions for each: Cu^II^ complexes are air stable and can be analyzed on the bench top, while Cu^I^ complexes are very air sensitive and required rigorous anaerobic conditions of a glovebox.

Additionally, both complexes were subjected to conditions with increasing concentrations of water in MeCN. The Cu^II/I^ couple for ^Pic^N4 was only shifted slightly to −0.800 V vs. Fc^0/+^ even in a 70% water to MeCN solution (with 0.2 M TBAP). The reversibility of the couple remained stable throughout the course of the experiment (Figure S17). The Cu^II/I^ couple of ^PicMe^N4 shifted less drastically to −0.600V vs. Fc^0/+^ after adding up to 70% water to an MeCN solution (with 0.2 M TBAP). The system was overall reversible but at higher concentrations of water, and additional oxidation and reduction peaks appeared probably due to water binding to the metal center over MeCN (Figure S18).

### 2.4. Ligand Acidity Constants and Complex Stability Constants

**Figure 4.**
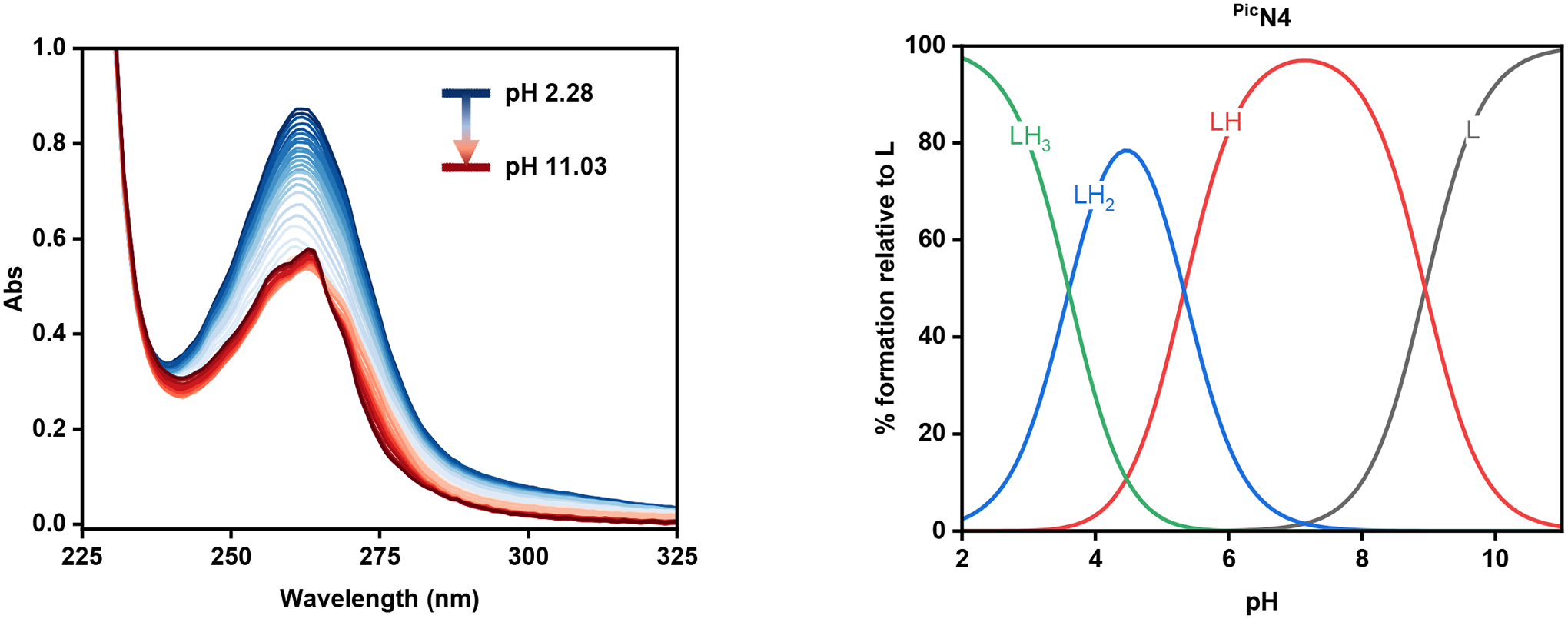
Variable pH (2.28–11.03) UV-Vis spectra of ^Pic^N4 in 0.1 M KCl at 25 °C (**left**) and its species distribution plot (**right**). [^Pic^N4]_tot_ = 60 μM.

To determine the acidity constants (pKa) of ^Pic^N4 and ^PicMe^N4, UV-Vis spectrophotometric titrations were performed and the changes in the spectra were monitored. To a solution of either ^Pic^N4 or ^PicMe^N4 in 0.1 M KCl, aliquots of 0.15 M KOH were added and the UV-Vis spectra were recorded at each pH. For the ^Pic^N4 ligand, the increase of the solution’s pH results in the steady decrease of the π—π* transition band at 264 nm, until around pH 7, at which point the absorbance begins to increase (Figure 4). The data was then simulated in the HypSpec 2014 program (Protonic Software, UK) [32], which afforded the species distribution plot (Figure 4) and three pKa values: 8.94, 5.32, and 3.60. These values are tentatively assigned to the tertiary amine nitrogen, pyridine on the N4 backbone, and picolyl nitrogen, respectively. Despite containing six potential sites for protonation, only three pKa values were determined. This is likely due to the increased electrostatic repulsion that occurs upon sequential protonation steps, making it difficult to observe higher charged species in the pH range of the titration [33].

For the ^PicMe^N4 ligand, a similar decrease in the peak at 261 nm occurred upon the increase in pH, until the lowest absorbance was observed at approximately pH 4 (Figure S25). Thereafter, the absorbance was observed to increase until a plateau at pH 7. Analysis using HypSpec provided four pKa values, the highest of which, 11.13, is assigned to the deprotonation of the methyl amine nitrogen (Table 4). The next highest value, 9.16, is assigned to the deprotonation of the tertiary amine amended with the 2-methylpyridine arm, which has previously been shown to lower the basicity of amine nitrogens attached to it [34–36]. This assignment also aligns with the highest pKa observed in ^Pic^N4, which contains two similar amine sites.

**Figure 5.**
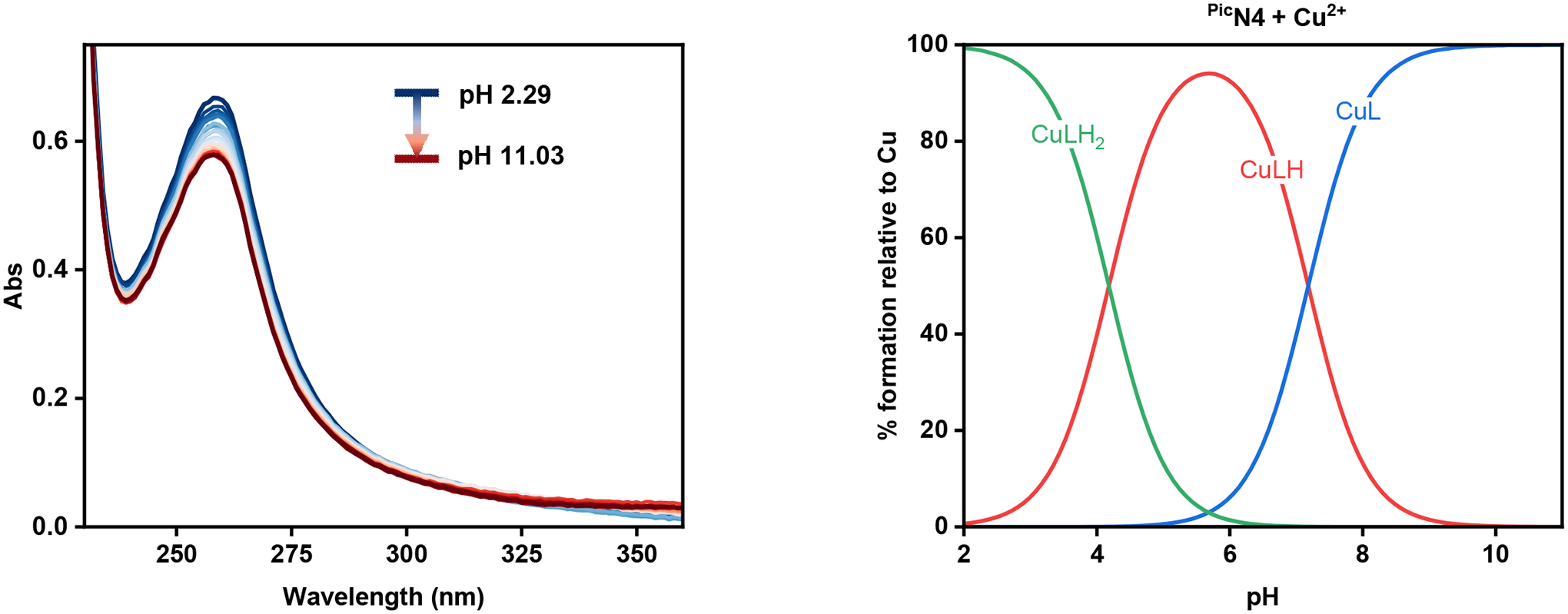
Variable pH (2.29–11.03) UV-Vis spectra of the ^Pic^N4 + Cu^2+^ system in 0.1 M KCl at 25 °C (**left**) and its species distribution plot (**right**). [Cu^2+^]_tot_ = [^Pic^N4]_tot_ = 50 μM.

To obtain the Cu^II^ stability constants for the complexes, similar spectrophotometric pH titrations were performed for a 1:1 mixture of Cu^2+^ and ligand in 0.1M KCl (Figure 5). Analysis of the spectral changes occurring in the UV for each complex gave a series of stability constants, as summarized in Table 5. The log(K_Cu(II)L_) values reveal that ^Pic^N4 is able to form slightly more stable copper complexes than ^PicMe^N4. In the case of ^PicMe^N4, a value corresponding to the deprotonation of water was also obtained (7.75), but this was not observed for ^Pic^N4. This could likely be attributed to the open coordination site available for ^PicMe^N4, as evidenced in the crystal structure, which would allow for the binding and subsequent deprotonation of a water molecule.

The determination of the Cu^I^ stability constants for each complex relied on the Cu^II^ stability constant and the E_1/2_ values determined in the aqueous CV experiments. CVs of the complexes in aqueous conditions with 0.1 M NaOAc as a supporting electrolyte revealed E_1/2_ values of −0.415 V and −0.220 V vs. Ag/AgCl for **1**^**2+**^ and **3**^**2+**^, respectively (Figures S19 and S20). It is worth noting that the aqueous CV data of **1**^**2+**^ showed an additional reversible redox couple at −0.245 V vs. Ag/AgCl, which could be attributed to an alternative coordination mode, perhaps from the binding of an acetate ion present in solution. Another possible explanation for the observation of two species in solution could be two protonation states, as the species distribution for this complex shows nearly equal amounts of CuL and CuLH species at pH 7. Nevertheless, for the purpose of determining Cu^I^ stability constants, the more negative reduction potential was used in the Nernst equation. Stability constants for Cu^I^ could then be obtained by applying a Nernstian relationship using the reduction potentials, which results in log(K_Cu(I)L_) values of 7.05 and 9.46 for ^Pic^N4 and ^PicMe^N4, respectively.

Another important consideration for ^64^Cu chelators is the possibility of transmetalation with biogenic metals in vivo. Therefore, the stability constants of the ligands ^Pic^N4 and ^PicMe^N4 towards Zn^2+^ were also determined. Both ligands have significantly lower Zn stability constants as compared to those for Cu, and the affinity towards Zn is also markedly lower at biological pH. This data indicates that the copper complexes of ^Pic^N4 and ^PicMe^N4 are unlikely to undergo transmetalation with zinc, a promising trait for potential ^64^Cu chelators.

When comparing the log(K_Cu(II)L_) to other commonly used ^64^Cu chelators, it is observed that ^Pic^N4 and ^PicMe^N4 have moderately lower stability constants than the other chelators (Table 6). Many of these chelators have N,O-based donor sets, but notably also have irreversible reduction potentials (e.g. DOTA, TETA). The chelators described in this work have the added benefit of reversible Cu^II/I^ redox couples, allowing for the ability to form stable Cu^I^ complexes.

### 2.5. Radiolabeling Studies

**Figure 6.**
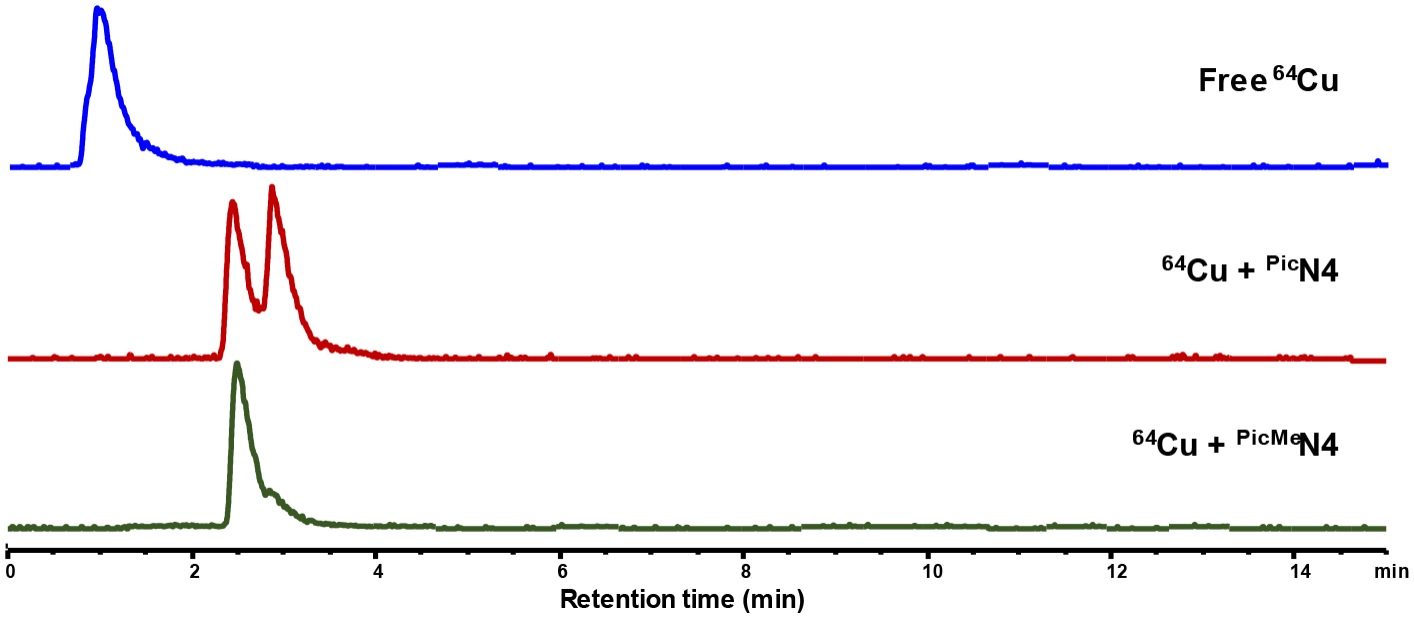
Radio-HPLC chromatograms for the ^64^Cu labeled complexes of ^Pic^N4 and ^PicMe^N4.

The radiolabeling capabilities of ^Pic^N4 and ^PicMe^N4 were also evaluated. Using a stock solution of ^64^CuCl_2_ diluted in ammonium acetate buffer (pH 5.5), mixtures of the ligands and ^64^CuCl_2_ were incubated at 45 °C for 30 min. These relatively mild conditions are comparable to those used for the common ^64^Cu chelators like NOTA and DOTA [38]. The radiolabeled compounds were then analyzed by radio-HPLC using water (0.1% TFA) and acetonitrile (0.1% TFA) as the mobile phase with a gradient of 0–100% acetonitrile over 15 min (Figure 6). A control of only ^64^CuCl_2_ in ammonium acetate was also analyzed to compare the retention times. Both ligands showed complete conversion to ^64^Cu complexes, with no remaining free ^64^Cu being observed in either radio-HPLC trace. While ^PicMe^N4 shows one peak in the chromatogram, ^Pic^N4 shows two peaks close to one another. One possible explanation for this observation is an alternative coordination environment around the copper center, such as a different counterion (e.g. chloride, acetate) bound to the metal center. This observation is consistent with the aqueous CV studies that show ^Pic^N4 also having two species present in solution (Figure S19).

After confirming that the ligands were able to be radiolabeled with ^64^Cu, the lipophilicity of the complexes was determined by measuring the octanol/PBS partition coefficient (logD_oct_, Table 7). Both dicationic complexes are particularly hydrophilic, with ^64^Cu-^PicMe^N4 having a more negative partition coefficient than ^64^Cu-^Pic^N4.

### 2.6. Cytotoxicity Studies

**Figure 7.**
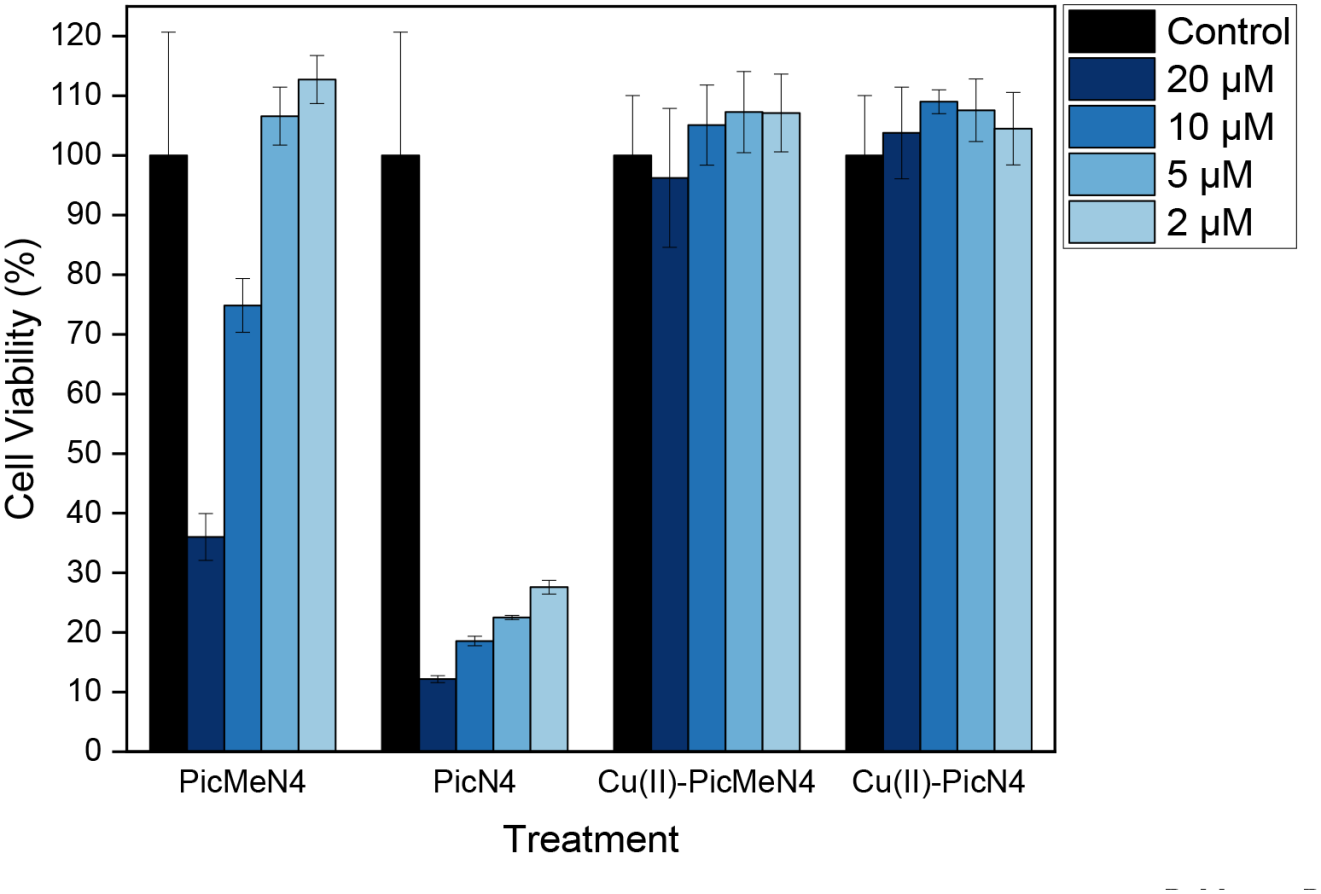
Cell viability (% of control) of Neuro2A cells upon incubation with ^PicMe^N4, ^Pic^N4, and their Cu^II^ complexes at 2, 5, 10, and 20 μM concentrations.

In order to test the plausibility of in vivo applications for these two chelators, cytotoxicity studies were performed using an Alamar blue assay on mouse neuroblastoma Neuro2a (N2a) cells. Cells were treated with each compound of their Cu^II^ complexes and cell viability was evaluated after a 48 h incubation period. The percentage of cell viability, as summarized in Figure 7, revealed that both ligands ^Pic^N4 and ^PicMe^N4 are toxic at higher concentrations, but ^PicMe^N4 is significantly less toxic than ^Pic^N4. Notably, the addition of Cu greatly reduces the toxicity of these ligands, with both complexes showing extremely high cell viability across the board, even at concentrations of 20 μM.

## 3. Conclusions

Inspired by previous pyridinophane ligands, herein we report two new ligand systems, ^Pic^N4 and ^PicMe^N4. The 2-methylpyridyl arms of these ligands bind to the Cu center in place of exogenous ligands and allow for a polydentate binding mode greater than ^tBu^N4. The hexadentate ^Pic^N4 ligand offers the metal center a fully bound, distorted octahedral geometry, which can shield the metal center from side reactions. The asymmetric ^PicMe^N4 ligand offers five coordinating atom donors with the option of binding one exogenous ligand. This flexible pentadentate ligand can adopt geometries other than distorted octahedral and could be used to probe electrocatalytic transformations.

When bound to copper, ^Pic^N4 and ^PicMe^N4 both stabilized Cu^II^ and Cu^I^ centers, which were characterized by various spectroscopic means. Crystal structures were also obtained for all four compounds, showing a preferred geometry of distorted octahedral for the Cu^II^ complexes and a distorted square pyramidal geometry for the Cu^I^ complexes. Electrochemically, ^Pic^N4 exhibits a reversible Cu^II/I^ couple at a low potential of −0.1 V vs. SHE. Conversely, the ^PicMe^N4 Cu^II/I^ couple was also reversible, but the ability to bind an exogenous ligand caused other redox features to appear. Both Cu^I^ and Cu^II^ stability constants for each ligand were also determined, as were their affinities for Cu^II^ at biologically relevant pHs.

## Supplementary Material

Supp Info

## Figures and Tables

**Table 1.** Selected bond distances (Å) and angles (°) of cations **1**–**4**.

	1^2+^	2^+^	3^2+^	4^+^
**Cu-N1**	2.056(4)	2. 1341(1)	1.944(9)	2.1286(1)
**Cu-N2**	2.028.(4)	2.0817(1)	2.173(7)	2.0768(2)
**Cu-N3**	2.003(4)	1.9640(1)	1.967(8)	1.9461(1)
**Cu-fE4**	2.276(4)	2.3983(1)	2.258(4)	2.P957(2)
**Cu-N5**	2.348(4)	2.3456(1)	2.165(8)	2.262(2)
**Cu-N6**	2.017(4)	3.323	2.219(9)	---
**N2-Cu-N1**	84.43	81.36	82.9	83 .07
**N4-Cu-N5**	148.38	146.78	152.1	147.85
**φ (°)** ^a^	86.60, 84.19	86.91, 87.93	88.07, 89.28	88.19, 8700
**θ (°)** ^b^	36.19	28.92	20.39	31.30

a φ (°) designates the angles between the average plane of two pyridine rings and a mean equatorial plane;

b θ (°) designates the angle between the equatorial plane made between atoms N1, N2, and Cu and the plane made between atoms N3, Cu, and N6 for Cu^II^ complexes and N3, Cu, and para-carbon on picolyl arm for Cu^I^.

**Table 2.** Selected EPR Data for Paramagnetic Complexes.

	g_x_	g_y_	g_z_	A_z_ (G)
**1·(OTf)**_**2**_	2.070	2.055	2.259	145
**3·(OTf)**_**2**_	2.067	2.056	2.264	152

**Table 3.** Selected Physical Parameters of Complexes **1**–**4**.

1^2+^	2^+^	3^2+^	4^+^
**E, V (ΔE**_**p**_ **, mV)** ^a,b,c^			
E_1/2_ = −0.752 (97) E_ox_ = 1.047	E_1/2_ = −0.716 (176)	E_1/2_ = −0.268 (E05) E_ox_ = 1.552	E_1/2_ = −0.441 (96)
**UV-Vis, λ**_**max**_**, nm (ε, M**^**−1**^ **cm**^**−1**^**), MeCN**			
257 (22,775), 340 (446), 717(146)	250 (8395), 362 (2438), 444 (893)	258 (12,251), 322 (671), 687 (91)	246 (11,731), 332 (3331), 370 (3854), 435 (9611)
**μ**_**eff**_ **(μ**_**B**_**) at 293 K, Evans’ Method, CD**_**3**_**CN**			
1.80	N/A	1.71	N/A

a Redox Potentials (vs. Fc/Fc+), 0.1 M tBAP/MeCN, 0.01 M Ag/AgNO_3_ or Ag wire reference, Δep is the separation between anodic and cathodic waves in mV, measured at 100 mV/s.

b **1**^**2+**^ & **3**^**2+**^ had 3-segment sweep.

c **2**^**+**^ & **4**^**+**^ had 5-segment sweep. N/A: not applicable.

**Table 4.** Acidity constants (pKa) of ligands.

	^Pic^N4	^PicMe^N4
**[H**_**4**_**L]**^**4+**^ **= [H**_**3**_**L]**^**3+**^ **+ H**^**+**^	-	2.47(9)
**[H**_**3**_**L]**^**3+**^ **= [H**_**2**_**L]**^**2+**^ **+ H**^**+**^	3.60(3)	5.46(9)
**[H**_**2**_**L]**^**2+**^ **= [HL]**^**+**^ **+ H**^**+**^	5.32(0)	9.16(9)
**[HL] = [L] + H** ^**+**^	8.94(6)	11.13(8)

**Table 5.** Stability constants (logK values) and calculated pM values for Cu and Zn complexes. Errors reported for the last digit.

	^Pic^N4 + Cu^2+^	^PicMe^N4 + Cu^2+^	^Pic^N4 + Zn^2+^	^PicMe^N4 + Zn^2+^
**M**^**2+**^ **+ H**_**2**_**L**^**+**^ **= [MH2L]**^**4+**^	4.13(3)	-	-	-
**M**^**2+**^ **+ HL**^**+**^ **= [MHL]**^**3+**^	7.40(1)	4.54(1)	2.67(2)	9.28(7)
**M**^**2+**^ **+ L = [ML]**^**2+**^	**17.96(3)**	**17.07(1)**	**11.45(4)**	**10.41(7)**
**[ML(H**_**2**_**O)]**^**2+**^ **= [ML(OH)]**^**+**^ **+ H**^**+**^	-	7.75(2)	-	-
**pM**^**2+**^ **(pH 7.4)**^a^	16.81	12.90	8.87	8.02
**log(K**_**Cu(II)L**_ **)**	17.96	17.07	-	-
**log(K**_**Cu(I)L**_ **)**	7.05	9.46	-	-

a Values calculated as −log[M]_free_, where [M^2+^] = 10^−6^ M, [L] = 10^−5^ M.

**Table 6.** Comparison of log(K_Cu(II)L_) values of commonly used ^64^Cu chelators.

Chelator	Log(K_Cu(II)L_)	Ref.
^Pic^N4	17.96	This work
^PicMe^N4	17.07	This work
YW-15-Me	14.7	[7]
DO4S	19.6	[16]
PCTA	19.1	[37,38]
EDTA	19.2	[37,39,40]
TETA	21.1	[39–42]
DOTA	22.2	[39–41]
cyclen	24.6	[39,40]

**Table 7.** Molecular weight and measured LogD values of the ^64^Cu complexes.

^64^Cu Complex	MW (g/mol)	log Doct
^**64**^ **Cu-** ^**Pic**^ **N4**	556.98	−1.564 ± 0.26
^**64**^ **Cu-** ^**PicMe**^ **N4**	479.90	−2.171 ± 0.09

## Data Availability

All research data can be found in the Supplementary Materials or can be requested from the corresponding author.
