# Supplementary material for: Pentadentate and Hexadentate Pyridinophane Ligands Support Reversible Cu(II)/Cu(I) Redox Couples
Source: Inorganics (Basel). Author manuscript; Available in PMC 2024 Sep 19. (PMC11412068; doi:10.3390/inorganics11110446)
Supplement: Supp Info [file NIHMS2022023-supplement-Supp_Info.pdf]

Supplementary Materials for

# Pentadentate and Hexadentate Pyridinophane Ligands Support Reversible Cu(II)/Cu(I) Redox Couples

Glenn Blade[1], Andrew J. Wessel[2], Karna Terpstra[1], and Liviu M. Mirica[1] *

[1] Department of Chemistry, University of Illinois Urbana-Champaign, 600 S. Matthews Ave, Urbana, Illinois 61801

[2] Department of Chemistry, Washington University, One Brookings Drive, St. Louis, Missouri 63130-4899.

*Correspondence to: mirica@illinois.edu

**Table of Contents**

| | | |
|---|---|---|
| **I.** | **Experimental details** | **S2** |
| **II.** | **NMR studies** | **S9** |
| **III.** | **Cyclic voltammetry studies** | **S23** |
| **IV.** | **UV-vis studies** | **S26** |
| **V.** | **pKa and stability constant determination** | **S26** |
| **V.** | **X-ray structure determination** | **S34** |
| **VI.** | **References** | **S46** |

## I. Experimental details

### General specifications

All manipulations were carried out under a nitrogen atmosphere using standard Schlenk and glovebox techniques if not indicated otherwise. All reagents for which the synthesis is not given were commercially obtained from Sigma-Aldrich, Acros, or Strem, and were used as received without further purification. Solvents were purified to use by passing through a column of activated alumina using an MBRAUN SPS stored over 3 Å molecular sieves. $^{H}$N4, $^{TsH}$N4, and $^{MeH}$N4 were prepared per literature procedures.[1i] Anhydrous samples of metal triflates from STREM were used for the synthesis of the corresponding metal complexes or prepared by literature procedures.[2] $^{1}$H-NMR (300.1 MHz) and $^{13}$C-NMR (75.1 MHz) spectra were recorded on a Varian Mercury-300 spectrometer, Varian Unity Inova-500, or a Varian Unity Inova-600 spectrometer. Solution magnetic susceptibility measurements were obtained by the Evans' method using coaxial NMR tubes at 293K.[3] Diamagnetic correction were applied as previously described.[4-5] UV-vis spectra were recorded on a Varian Cary 50 Bio spectrophotometer and are reported as $\lambda_{max}$, nm (ε, $M^{-1}$ $cm^{-1}$). EPR spectra were recorded on a JEOL JES-FA X-band (9.2GHz) EPR spectrometer at 77 K. EPR spectra simulation and analysis were performed using Bruker WINEPR SimFonia program, version 1.25. ESI-MS experiments were performed using a Thermo FT or Bruker Maxis Q-TOF mass spectrometer with an electrospray ionization source. Elemental analyses were carried out by Intertek Pharmeceutical Service. Cyclic voltammetry (CV) experiments were performed with a BASi EC Epsilon electrochemical workstation or a CHI 660D Electrochemical Analyzer. Electrochemical-grade $Bu_4NClO_4$ (Fluka) was used as the supporting electrolyte. Electrochemical measurements were performed under a blanket of nitrogen, and the analyzed solutions were sparged by purging with nitrogen. A glassy carbon disk electrode (d = 1.6 mm) was used as the working electrode, and either a Ag/AgCl wire electrode or a non-aqueous reference electrode (BASi) was used as the reference electrode. The reference electrodes were calibrated against ferrocene, $Cp_2Fe$ (Fc).

### X-ray Structural Determination

Suitable crystals were mounted on MiTeGen cryoloops in random orientations in a Bruker Kappa Apex-II CCD X-ray diffractometer equipped with an Oxford Cryostream LT device and a fine focus Mo Kα radiation X-ray source (λ = 0.71073 Å). Preliminary unit cell constants were determined with a set of 36 narrow frame scans. Typical data sets consist of combinations of v and f scan frames with a typical scan width of 0.5° and a counting time of 15−30 s/frame at a crystal-to-detector distance of 3.5 cm. The collected frames

were integrated using an orientation matrix determined from the narrow frame scans. Apex II and SAINT software packages (Bruker Analytical X-Ray, Madison, WI, 2008) were used for data collection and data integration. Analysis of the integrated data did not show any decay. Final cell constants were determined by global refinement of xyz centroids of reflections from the complete data sets. Collected data were corrected for systematic errors using SADABS Bruker Analytical X-Ray, Madison, WI, 2008) based on the Laue symmetry using equivalent reflections. Crystal data and intensity data collection parameters are listed in Tables S4-S11. Structure solutions and refinement were carried out using the SHELXTL-PLUS software package.[6-7] The structures were solved by direct methods and refined successfully. Full matrix least-squares refinements were carried out by minimizing $\Sigma w(Fo^2-Fc^2)^2$. The non-hydrogen atoms were refined anisotropically to convergence. The hydrogen atoms were treated using appropriate riding model. The crystallographic datasets have been deposited at CCDC under the record numbers 2049802, 2049803, 2049804, and 2049805.

**Acidity and Stability Constant Determination**

Spectrophotometric pH-titrations were performed for determination of the acidity constants for the ligands and the stability constants for their Cu(II) complexes. In general, Ligands (60 µM, 0.1 M KCl) or the Ligands + Cu(II) (60 µM, 0.1 M KCl), were titrated with small aliquots of 0.15 M KOH from low pH to high pH at room temperature until no more spectral changes (pH range 2-11). The starting low pH is achieved by addition of 0.5M HCl diluted in Chelex-treated water, until the desired starting pH is reached. About 50 UV−vis spectra were collected from the titration experiments, the corresponding acidity and stability constants were then calculated using the HypSpec computer program (Protonic Software, UK), a commonly used method for studying metal complexes.[8]

The ligand-Cu(I) stability constant were calculated from half-wave potentials $E_f$ and the $Cu^{II}$ complex stability constants using the Nernst relationship.[9] For a reversible redox couple, $E_f$, were obtained by averaging the anodic and cathodic peak potentials. The value of the aqueous Cu(II/I) redox couple ($E^{\circ}_{aq}$), 0.230 V vs Ag/AgCl, was obtained from a control of $Cu(OTf)_2$ in the same aqueous conditions the complex CVs were measured in (1:1 MeCN:0.1M NaOAc in $H_2O$).

$$E^f = E^{\circ}_{aq} - \frac{2.30RT}{nF} \log\left(\frac{K_{Cu(II)L}}{K_{Cu(I)L}}\right)$$

$$n = 1, F = 96485.3329\ C \cdot mol^{-1}, R = 8.3145\ J \cdot mol^{-1} \cdot K^{-1}, T = 298.15\ K$$

**Cytotoxicity studies**

Mouse neuroblastoma Neuro2a (N2a) cell line was purchased from the American Type Culture Collection (ATCC CCL-131TM). Cells were grown in regular growth media for N2a cells (DMEM/10% FBS) in a 5% CO2 humid atmosphere at 37 °C. Cells were seeded in a 96-well plate at 1.6 x 105 cells/well in regular growth media. After 24 hour of incubation in a 5% CO2 humid atmosphere at 37 °C, media was replaced with DMEM-N2 media and incubated for 1 hr prior to addition of reagents. Cells were treated with $^{\text{Pic}}$**N4** and $^{\text{PicMe}}$**N4** and their corresponding $Cu^{II}$ complexes (≤0.5% DMSO was utilized to aid ligand/complex solubility). After 48 hours of in a 5% CO2 humid atmosphere at 37 °C, alamarBlueTM cell viability reagent (Thermo Fisher Scientific) was added to each well and incubated for 60 min at 37 °C. Fluorescence intensity was measured at 590 nm (excitation wavelength = 560 nm) on a SpectraMax M2e plate reader.

**Radiolabeling**

$^{64}Cu$ was produced by a (p,n) reaction on enriched $^{64}Ni$ on a TR-19 biomedical cyclotron (Cyclotron Corporation, Berkeley, CA) at Mallinckrodt Institute of Radiology, S5 Washington University School of Medicine, and purified with an automated system using standard procedures. Radiolabeling studies were performed at the Experimental Molecular Imaging Laboratory (EMIL) in the Beckman Institute for Advanced Science and Engineering at UIUC. A stock solution of $^{64}CuCl_2$ was diluted with a 10-fold excess of 0.1 M ammonium acetate ($NH_4OAc$) buffer (pH 5.5). Labeling of PicN4 and PicMeN4 with $^{64}Cu$ was achieved by adding 20 µL of 1 mM compounds to 7.4 MBq (200 µCi) of $^{64}CuCl_2$ in 100 µL of 0.1 M $NH_4OAc$ of pH 5.5. The resultant solution was allowed to react for 30 min at 60 °C with agitation (1000 rpm) on a thermomixer. Radiolabeled compounds were analyzed by high-performance HPLC, with water (0.1% TFA) and acetonitrile (0.1% TFA) mobile phase with a gradient of 0–100% acetonitrile over 15 min with a flow rate of 1 mL/min.

**LogD Determination of $^{64}$Cu-Labeled Complexes**

A 5 µL aliquot of $^{64}Cu$-labeled complexes (0.37 MBq, 10 µCi) was added to a two-phase system consisting of an organic *n*-octanol and aqueous buffer PBS of pH 7.4 (500 µL/each). The mixture was vortexed at 1000 rpm for 1 h to allow distribution of the radiolabeled compound between the two phases and then given 30 min without agitation for the layers to separate. Aliquots (100 µL) were withdrawn from aqueous and *n*-octanol layers, and the amount of radioactivity was counted in an automated γ counter. The distribution coefficient was quantified using the following equation of $\log D_{oct} = \log([M]_{oct}/[M]_{aq})$. The

experiment was conducted with five replicates, and the average of the different measurements was recorded as the final log $D_{oct}$ value for each compound.

## Syntheses of $^{Pic}$N4, $^{PicMe}$N4, and their Cu complexes

*Synthesis of $^{Pic}$**N4**[10]*

The $^{H}$N4 (1.18 g, 4.91 mmol) was suspended in diisopropylethylamine (4.00 mL, 23.0 mmol) and MeCN (200 mL) in a 500 mL round bottom flask. To this mixture, 2-(chloromethyl)pyridine hydrochloride (1.67 g, 10.2 mmol) and a catalytic amount of tetrabutylammonium bromide (0.020 g) was added and the solution stirred at reflux for two days. To work up, ice was added to the warm reaction solution, the solution basified with a small amount of potassium hydroxide to reach a pH of 13, and the aqueous layer was extracted with DCM (3 x 500 mL). The organic layer was dried with potassium carbonate, and the solvent removed by rotary evaporation. The resulting tan-white solid was dried well under vacuum. (1.77 g, 81%).

$\delta_H$(400 MHz, $CDCl_3$): 8.608 (d, *J* = 5.3 Hz, 2 H), 7.764 (m, *J* = 5.5 Hz, 4 H), 7.214 (d of d, *J* = 7.2 Hz, 2 H), 7.114 (t, *J* = 7.8 Hz, 2 H), 6.774 (d, *J* = 8.4 Hz, 4 H), 4.162 (s, 4 H), 3.964 (s, 8 H); $\delta_C$(75 MHz, $CDCl_3$): 159.659, 157.388, 149.234, 136.524, 135.352, 123.344, 122.553, 122.231, 65.762, 63.689; ESI-MS (m/z): 423.3246 [$^{Pic}$N4+1]$^+$, 445.3021 [$^{Pic}$N4+23]$^+$

*Preparation of [($^{Pic}$N4)Cu$^{II}$](OTf)$_2$: **1·(OTf)$_2$***

The $^{Pic}$N4 (51 mg, 0.12 mmol) and copper (II) triflate, Cu$^{II}$(OTf)$_2$, (43 mg, 0.12 mmol) were added to a 20 mL vial. MeCN (1 mL) was added to the solids and the color immediately changed from one white and one light green solids to a blue solution. The reaction was stirred overnight. The MeCN solution mixture was filtered through a 0.2 μm nylon syringe filter and set up for diethyl ether diffusion recrystallization at room temp over two days. When the large blue elongated crystals had formed, the pale yellow/orange solution was decanted away and the crystals rinsed with diethyl ether and pentane. The crystals were then dried under reduced pressure to remove excess solvent (78.2mg, 83.3%). ESI-MS (m/z): 242.5750 [M]$^{2+}$, Calc: 242.5752; EA: Found: C, 42.90%; H, 3.53%; N, 10.60%. Calc $C_{28}H_{26}CuF_6O_6N_6S_2$: C, 42.88%; H, 3.34%; N, 10.72%.; Evan's Method (MeCN): 1.7963 $\mu_B$

*Preparation of [($^{Pic}$N4)Cu$^{I}$]OTf: **2·OTf***

The $^{Pic}$N4 (0.24 g, 5.8 mmol) and tetrakisacetonitrile copper (I) triflate, [(MeCN)$_4$Cu$^{I}$]OTf, (0.22 g, 5.9 mmol) were added to a 20 mL vial. MeCN (3 mL) was added to the solids and the color immediately changed from two white solids to a deep orange/brown solution. The reaction was stirred for one hour. The solution was concentrated by vacuum to roughly 1 mL and the solution was split to make two sets of

recrystallizations: 1) 0.5 mL of concentrated MeCN solution and 3 mL of dry diethyl ether layered on top and 2) 0.5 mL of concentrated MeCN solution and 3 mL of dry toluene layered on top. These recrystallizations were placed in the -35 °C freezer for four days. The large, dark-orange crystals had formed on the bottom of the vial in each recrystallization. These were rinsed with ether and harvested (200.8 mg, 55%). $\delta_H$(300 MHz, $CDCl_3$): 8.769 (d, 2 H), 7.883 (t, 2 H), 7.556 (d, 2 H), 7.382 (t, 2 H), 7.330 (t, 2 H), 6.782 (d, 4 H), 4.621 (s, 4 H), 4.307 (d, 4 H), 3.875 (d, 4 H); EA: Found: C, 50.98%; H, 4.24%; N, 13.26%. Calc. $C_{27}H_{26}CuF_3O_3N_6S$: C, 51.04%; H, 4.13%; N, 13.25%. 2D NMR spectra shown in supplemental information.

*Synthesis of $^{TsPic}$N4*

[$S_N2$] The $^{TsH}$N4 (1.96 g, 4.97 mmol) was suspended in diisopropylethylamine (1.75 mL, 14.4 mmol) and MeCN (200 mL) in a 500 mL round bottom flask. To this mixture, 2-(chloromethyl)pyridine hydrochloride (1.12 g, 8.67 mmol) and a catalytic amount of tetrabutylammonium bromide (0.020 g) was added and the solution stirred at reflux for two days. To work up, ice was added to the warm reaction solution, the solution basified with a small amount of NaOH to reach a pH of 13, and the aqueous layer was extracted with DCM (3 x 500 mL). The organic layer was dried with potassium carbonate, and the solvent removed by rotary evaporation. The resulting tan solid was dried well under vacuum. (2.16 g, 89%).

[Reductive amination] The $^{TsH}$N4 (1.72 g, 4.35 mmol), $NaHB(OAc)_3$ (1.28 g, 5.7 mmol), and 120 mL of DCM added to a dried 250 mL round-bottom flask charged with about 1 g of activated molecular sieves. Using an addition funnel, 2-pyridine carboxaldehyde (435 µL, 4.57 mmol) dissolved in 80 mL of DCM was slowly dripped in. The reaction vial was left to stir under a nitrogen atmosphere at room temperature overnight. To the reaction solution, saturated sodium bicarbonate solution (250 mL) was added as the solution stirred vigorously. The solution was then filtered using a coarse frit funnel to remove the molecular sieves and the vessel rinsed well with excess DCM. The organic layer was then separated, dried with anhydrous potassium carbonate, and then the solvent removed under reduced pressure rotary evaporation The white/off-white powder was then dried on vacuum line for at least five hours and stored in a desiccator (1.59 g, 75.0%).

$\delta_H$(300 MHz, $CDCl_3$): 7.767 (d, $J$ = 7.3 Hz, 2 H), 7.640 (d, $J$ = 7.3 Hz, 2 H), 7.378 (m, $J$ = 8.1 Hz, 4 H), 7.248 (m, $J$ = 8.1 Hz, 2 H), 7.157 (d, $J$ = 7.9 Hz, 3 H), 6.8995 (d, $J$ = 7.8 Hz, 4 H), 4.437 (s, 4 H), 4.127 (s, 2 H), 3.856 (s, 4 H), 2.467 (s, 3 H); $\delta_C$(125 MHz, $CDCl_3$): 155.553, 154.772, 143.589, 137.205, 136.850, 135.996, 135.692, 129.965, 129.696, 127.277, 126.964, 123.237, 121.060, 56.541, 54.208, 21.505; ESI-MS (m/z): 486.1909 [$^{TsPic}$N4+1]$^+$.

*Synthesis of $^{PicH}$N4*

Under $N_2$, $^{TsPic}$N4 (2.98 g, 6.14 mmol) was dissolved in 90% sulfuric acid (65 mL). This mixture was refluxed at 100 °C for 2.5 hours. After cooling, the solution was diluted with DI water (50 mL). In an ice bath, NaOH solid was added to the solution to basify to bring the pH to 14. The resulting solution was extracted with DCM (3 x 600 mL). The combined organic layers were dried over anhydrous potassium carbonate, and filtered. The filtrate was concentrated to dryness to give a white solid (1.77 g, 87.2%). $\delta_H$(300 MHz, $CDCl_3$): 8.586 (d, *J* = 4.5 Hz, 2 H), 7.740 (m, *J* = 8.2, 4 H), 7.209 (m, *J* = 4.4 Hz, 1 H), 7.058 (t, *J* = 7.1 Hz, 2 H), 6.693 (d, *J* = 8.1 Hz, 2H), 6.478 (d, *J* = 7.3 Hz, 2 H), 4.182 (s, 2 H), 3.963 (s, 4 H), 3.905 (s, 4 H); $\delta_C$(125 MHz, $CDCl_3$): 159.348, 158.667, 136.374, 135.621, 120.611, 119.649, 55.892 54.588; ESI-MS (m/z): 166.5973 [$^{PicH}$N4+2]$^{2+}$, 332.1870 [$^{PicH}$N4+1]$^{+}$.

*Synthesis of $^{PicMe}$N4*

Under $N_2$, $^{PicH}$N4 (1.77 g, 5.34 mmol) was dissolved in concentrated formic acid (150 mL, 3.98 mol) and 40% formaldeyde solution (15 mL, 163 mmol). This mixture was refluxed at 110 °C for 24 hours. After 24 hours, the reaction mixture was removed from the heat and 15 mL of 1 M HCl solution was stirred in. The solution was stirred for an additional 30 minutes. The solution was then concentrated to dryness via rotary evaporation and the residue was basified using KOH. The basic aqueous fraction was extracted with DCM and organic layers were combined and dried with potassium carbonate. The organic layer was then concentrated to dryness to give a white residue. This residue was purified by hot heptane extraction (3 x 500 mL) or soxhlet extraction with heptane (500 mL). The solution was then concentrated by rotary evaporation to yield a white powder (1.41 g, 76.6%). $\delta_H$(300MHz, $CDCl_3$): 8.564 (d, *J* = 5.1 Hz, 2 H), 7.689 (m, *J* = 6.0 Hz, 4 H), 7.182 (t, *J* = 7.9 Hz, 3 H), 6.806 (t, *J* = 7.6 Hz, 4 H), 4.145 (s, 2 H), 4.001 (s, 4 H), 3.852 (s, 4 H), 2.694 (s, 3 H); $\delta_C$(125 MHz, $CDCl_3$): 159.682, 157.507, 157.094, 149.236, 136.492, 135.394, 123.313, 122.575, 122.450, 122.211, 66.164, 65.784, 63.698, 49.143; ESI-MS (m/z): 346.2029 [$^{PicMe}$N4+1]$^{+}$.

*Preparation of [($^{PicMe}$N4)Cu$^{II}$(MeCN)](OTf)$_2$:* ***3·(OTf)$_2$***

The $^{PicMe}$N4 (54.4 mg, 1.54 mmoles) and Cu$^{II}$(OTf)$_2$, (55.1 mg, 1.53 mmoles) were added to a 20 mL vial. MeCN (1 mL) was added to the solids and the color immediately changed from white solids to a green solution which faded to blue. The reaction was stirred for 4 hours. The solution mixture was then concentrated down to a blue-green solution. This solution was then layered with toluene and stored in the -35 °C freezer overnight. The solution was then decanted to leave a blue-green residue which was rinsed with pentane to yield a blue-green powder which was dried under reduced pressure (50.3 mg, 47%). ESI-MS (m/z): 204.0616 [$^{PicMe}$N4Cu(MeCN)]$^{2+}$, Calc: 204.0619, EA: Found: C, 39.32%; H, 3.19%; N, 9.84%. Calc $C_{23}H_{23}CuF_6N_5O_6S_2$: C, 39.07%; H, 3.28%; N, 9.90%. Evan's Method (MeCN): 1.71 $\mu_B$.

*Preparation of [($^{PicMe}$N4)Cu$^{I}$]OTf:* ***4·OTf***

The $^{PicMe}$N4 (0.298 g, 0.84 mmol) and [$(MeCN)_4Cu^{I}$]OTf (0.295 g, 0.78 mmol) were added to a 20 mL vial. MeCN (3 mL) was added to the solids and the color immediately changed from two white solids to a deep orange/brown solution. The reaction was stirred for one hour in the dark. The solution was concentrated by vacuum to roughly 1 mL and the MeCN solution was recrystallized by diethyl ether diffusion at -35 °C freezer. The large, dark-orange crystals had formed on the bottom of the vial in each recrystallization. These were rinsed with diethyl ether and harvested (34.2 mg, 77%). $\delta_H$(300MHz, $CDCl_3$): 8.854(d, 1 H), 7.922 (t, 1 H), 7.448 (m, 2 H), 7.365 (t, 2 H), 6.814 (d of d, 4 H), 4.468 (s, 2 H), 4.248 (d, 2 H), 4.040 (d, 2 H), 3.668 (d, 2 H), 3.318 (s, 3 H); EA: Found: C, 47.49%; H, 4.07%; N, 12.50%. Calc $C_{22}H_{23}CuF_3N_5O_3S$: C, 47.35%; H, 4.15%; N, 12.55%.

## II. NMR studies

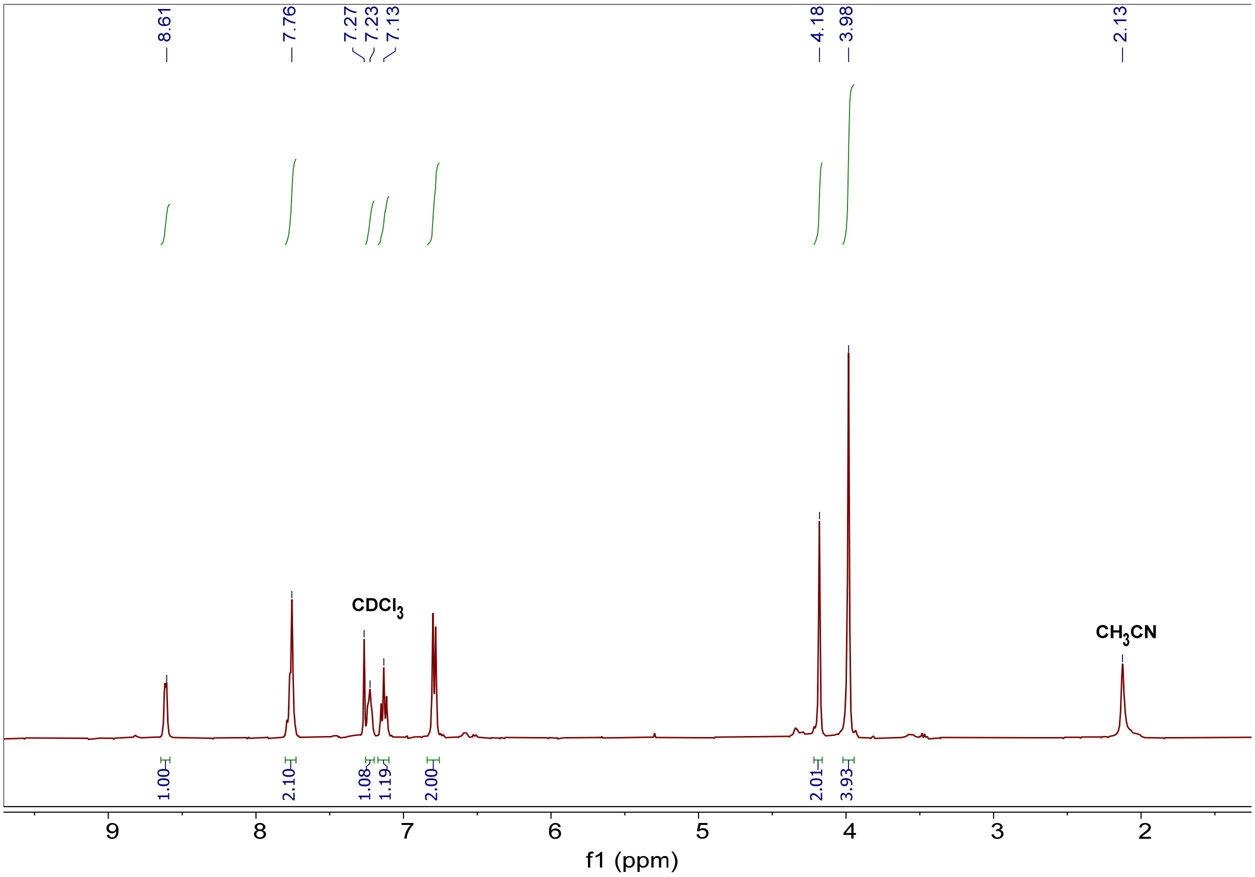


***Figure S1***. 300 MHz $^{1}$H-NMR spectrum of $^{Pic}$N4 in $CDCl_3$.

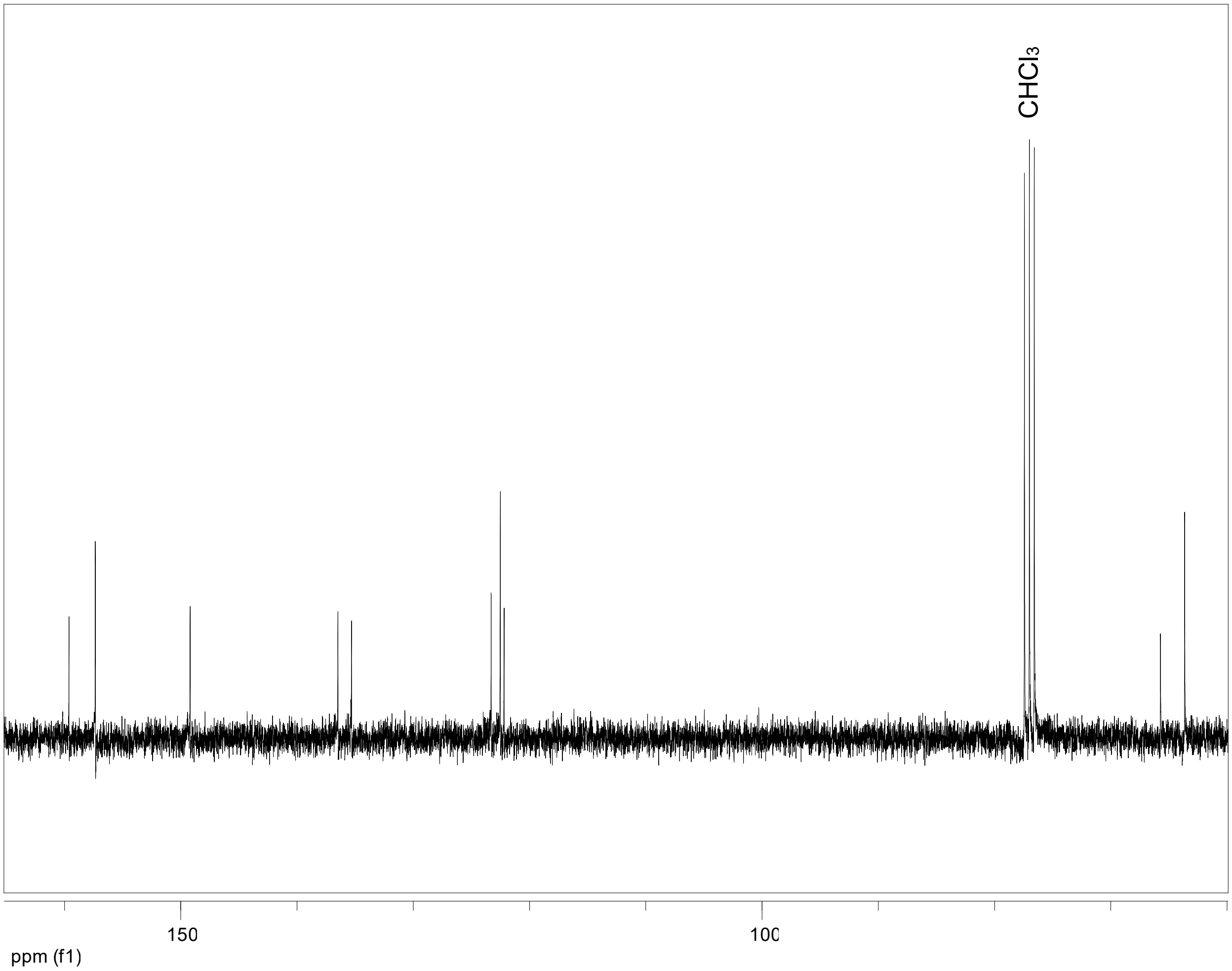


***Figure S2***. 125 MHz $^{13}$C-NMR spectrum of $^{Pic}$N4 in $CDCl_3$.

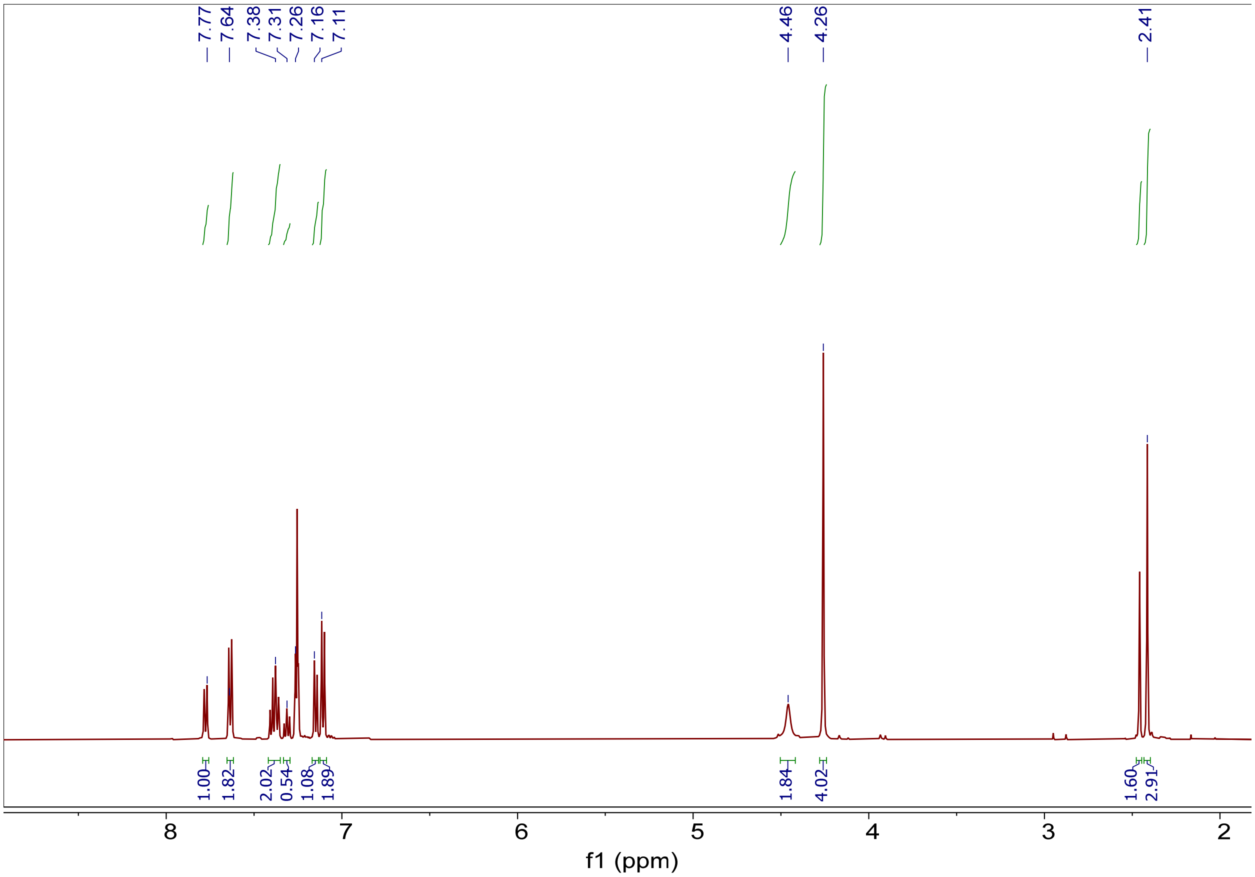


***Figure S3***. 600 MHz $^{1}$H-NMR spectrum of $^{PicTs}$**N4** in $CDCl_3$.

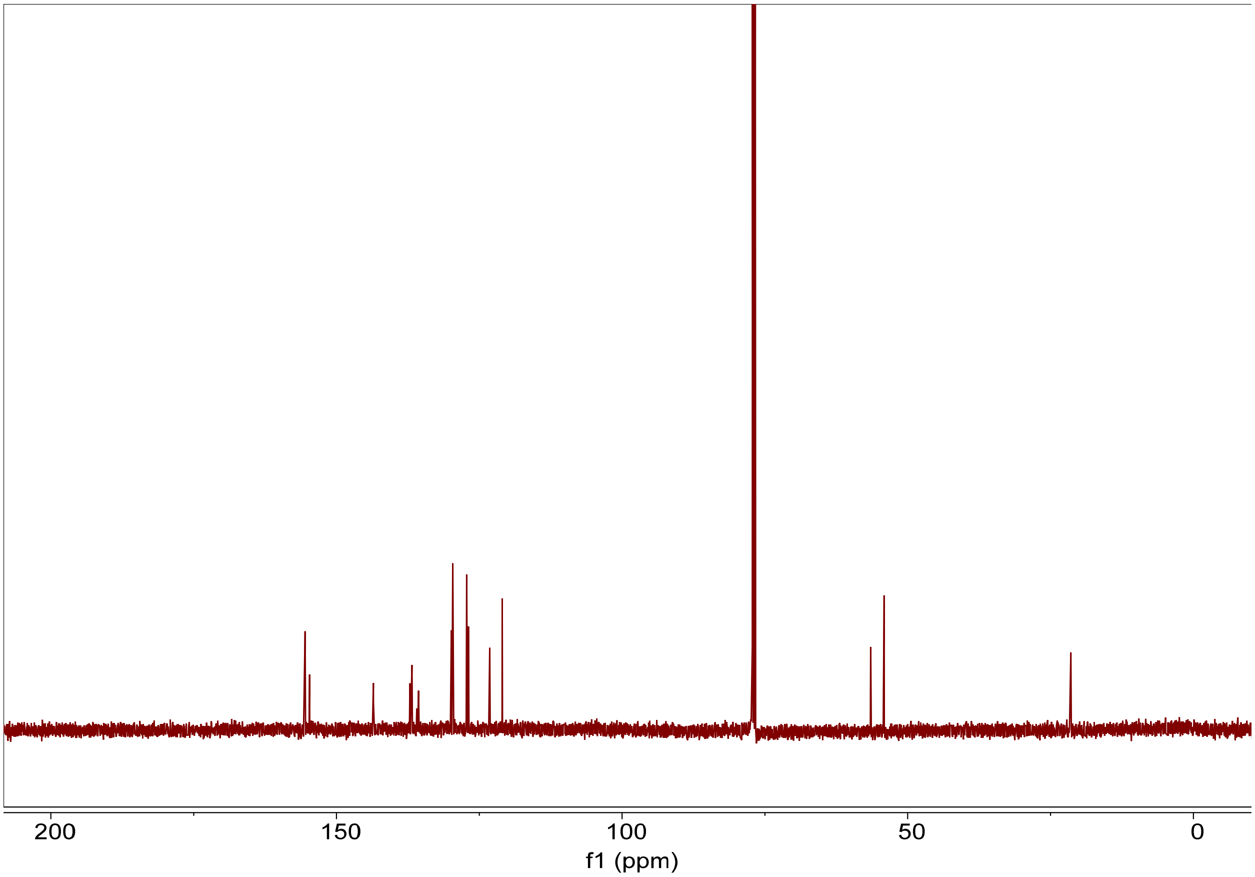


***Figure S4***. 150 MHz $^{13}$C-NMR spectrum of $^{PicTs}$**N4** in $CDCl_3$.

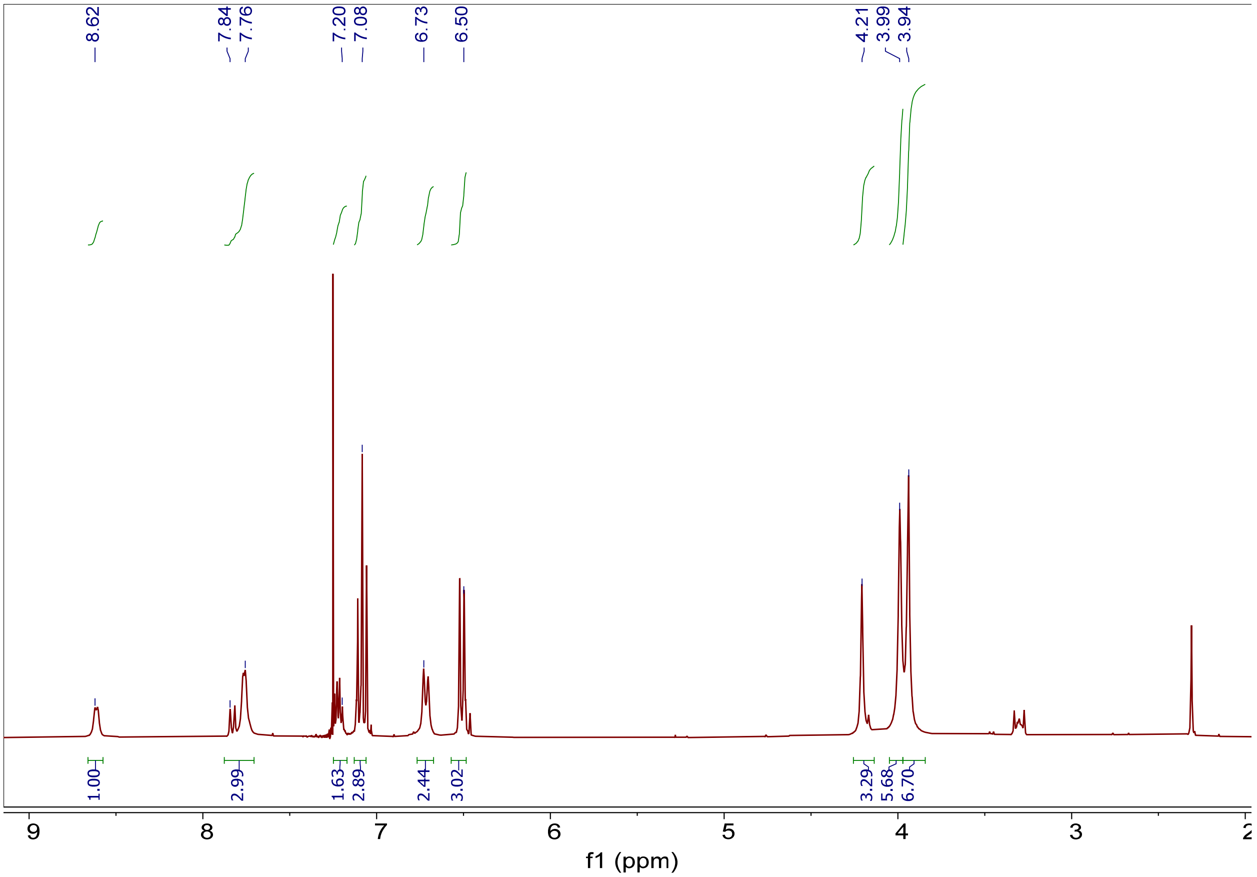


***Figure S5***. 600 MHz $^1$H-NMR spectrum of $^{PicH}$**N4** in $CDCl_3$.

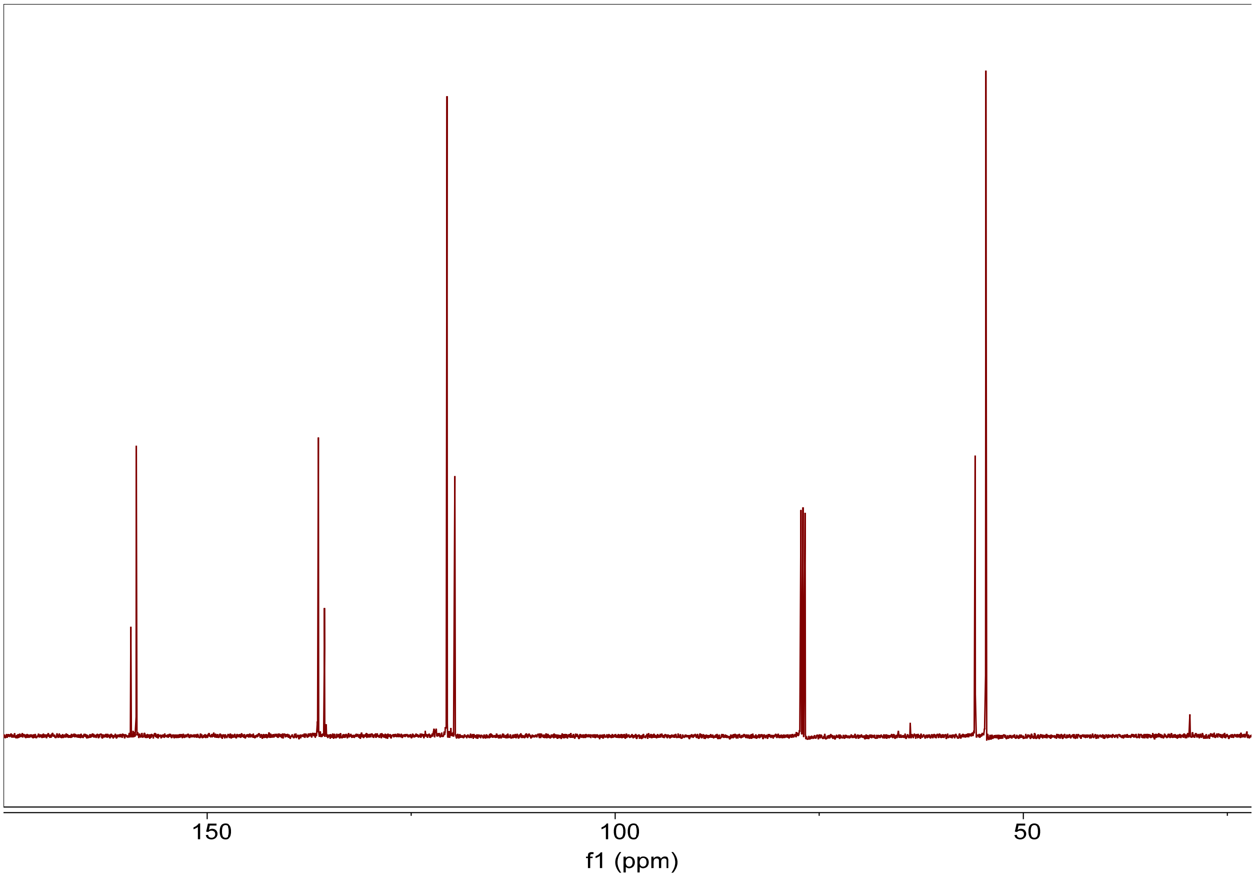


***Figure S6***. 150 MHz $^{13}$C-NMR spectrum of $^{\mathbf{PicH}}$**N4** in $CDCl_3$.

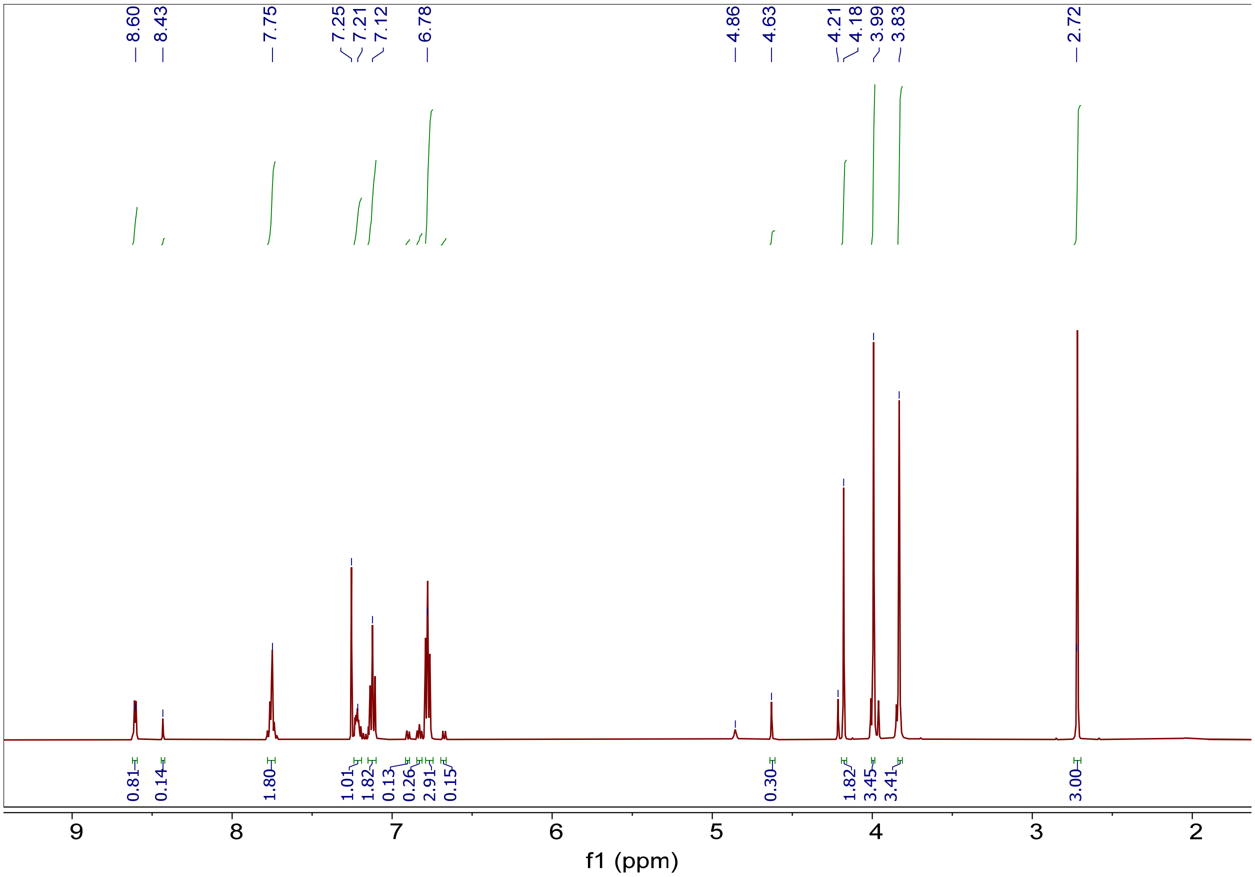


***Figure S7***. 600 MHz $^1$H-NMR spectrum of $^{PicMe}$**N4** in $CDCl_3$. Small amount of protonated $^{PicMe}$**N4** present in sample.

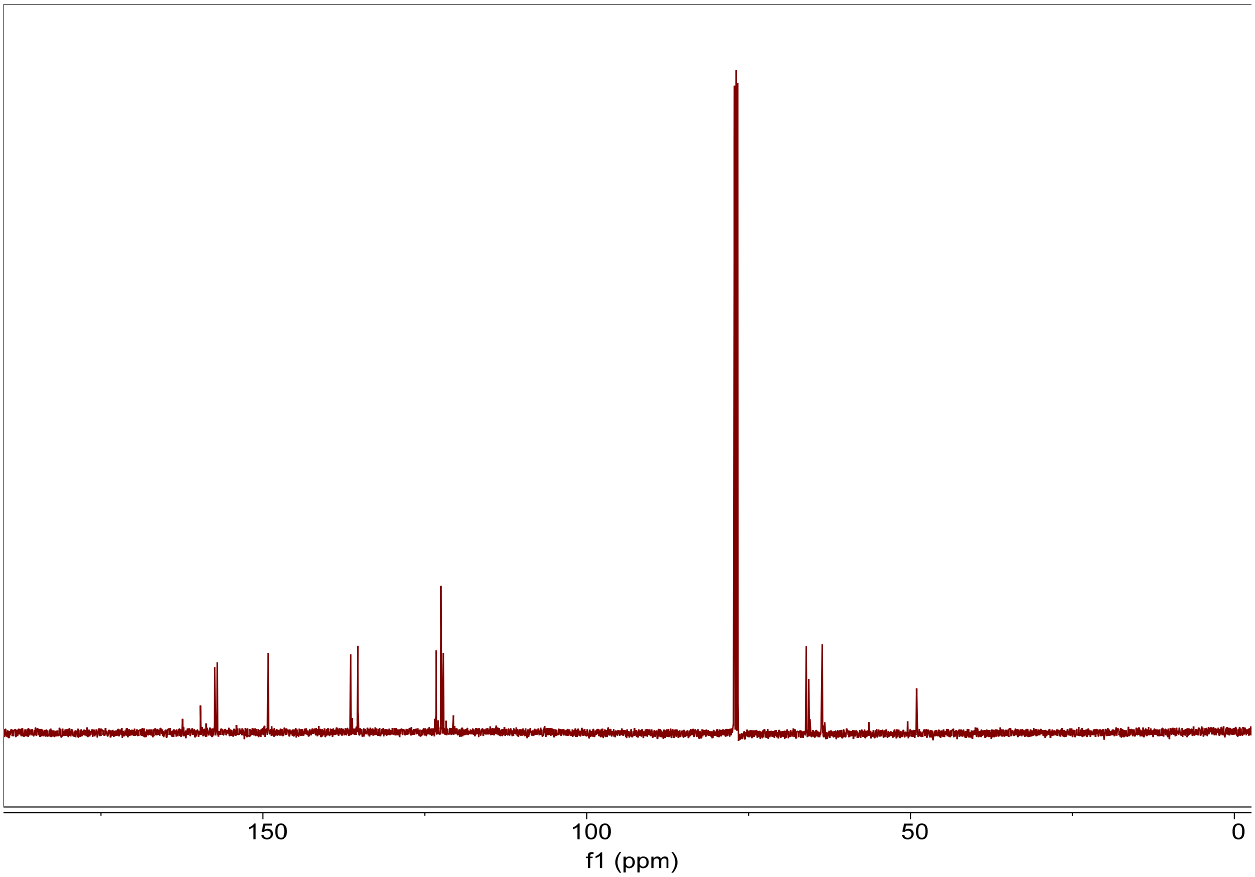


***Figure S8.*** 150 MHz $^{13}$C-NMR spectrum of $^{\mathbf{PicMe}}$**N4** in $CDCl_3$.

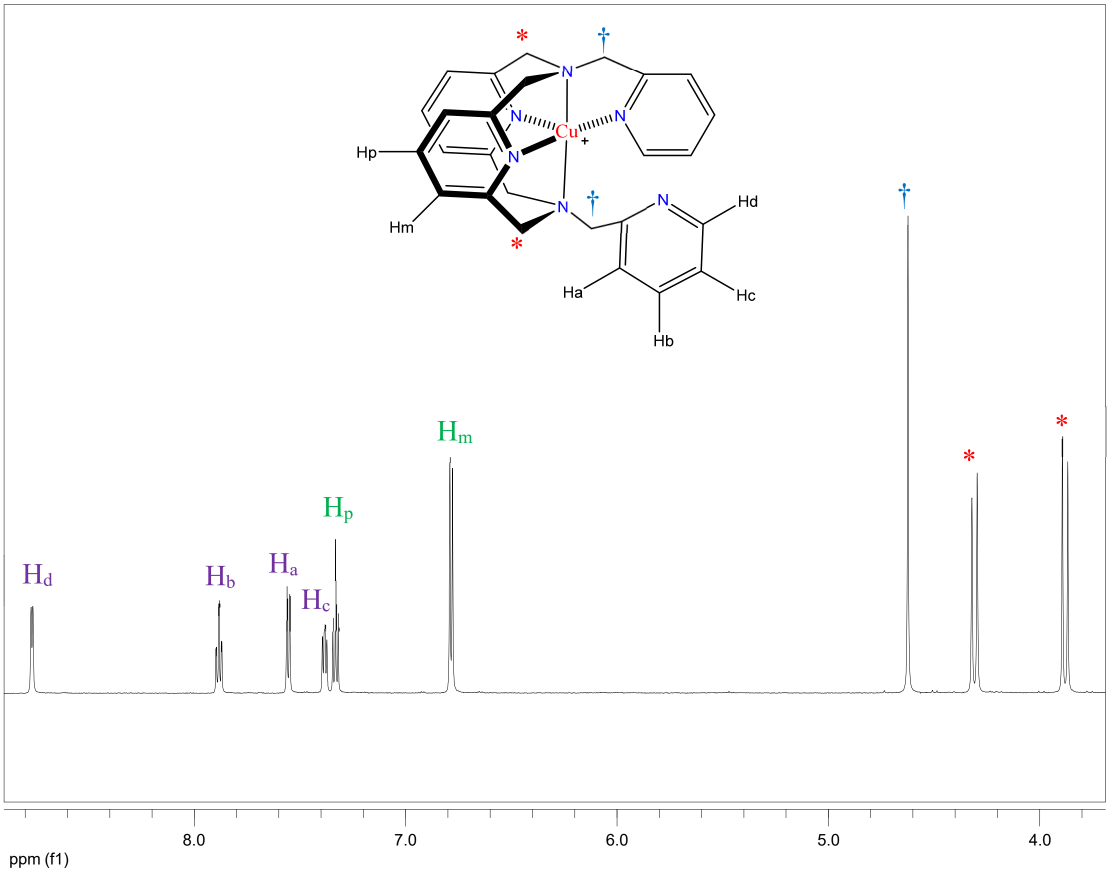


***Figure S9***. 600 MHz $^{1}$H-NMR spectrum of [($^{Pic}$N4)Cu$^{I}$](OTf) in $CD_3CN$ with assignments. * corresponds with $CH_2$ on the N4 backbone. † corresponds with $CH_2$ on the picolyl arm.

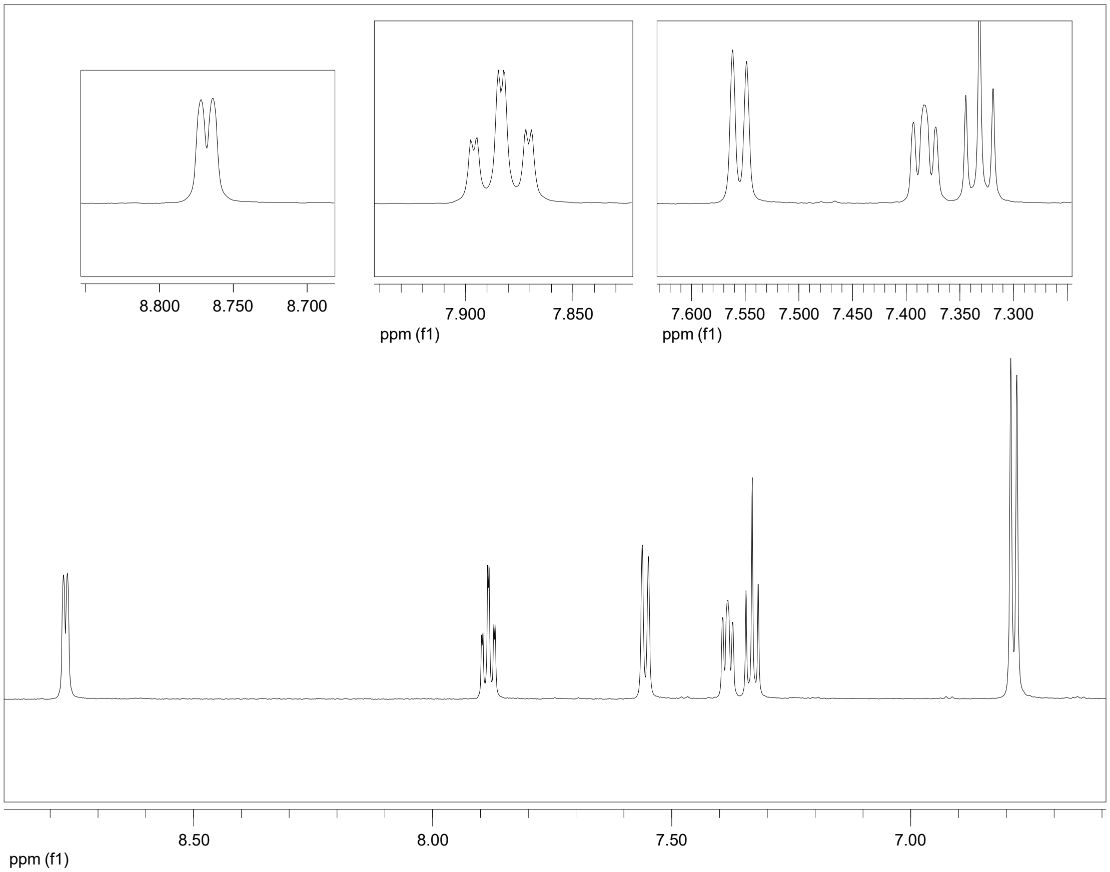


***Figure S10***. Expanded aromatic region of 600 MHz $^1$H-NMR spectrum of [($^{Pic}$N4)Cu$^I$](OTf) in $CD_3CN$.

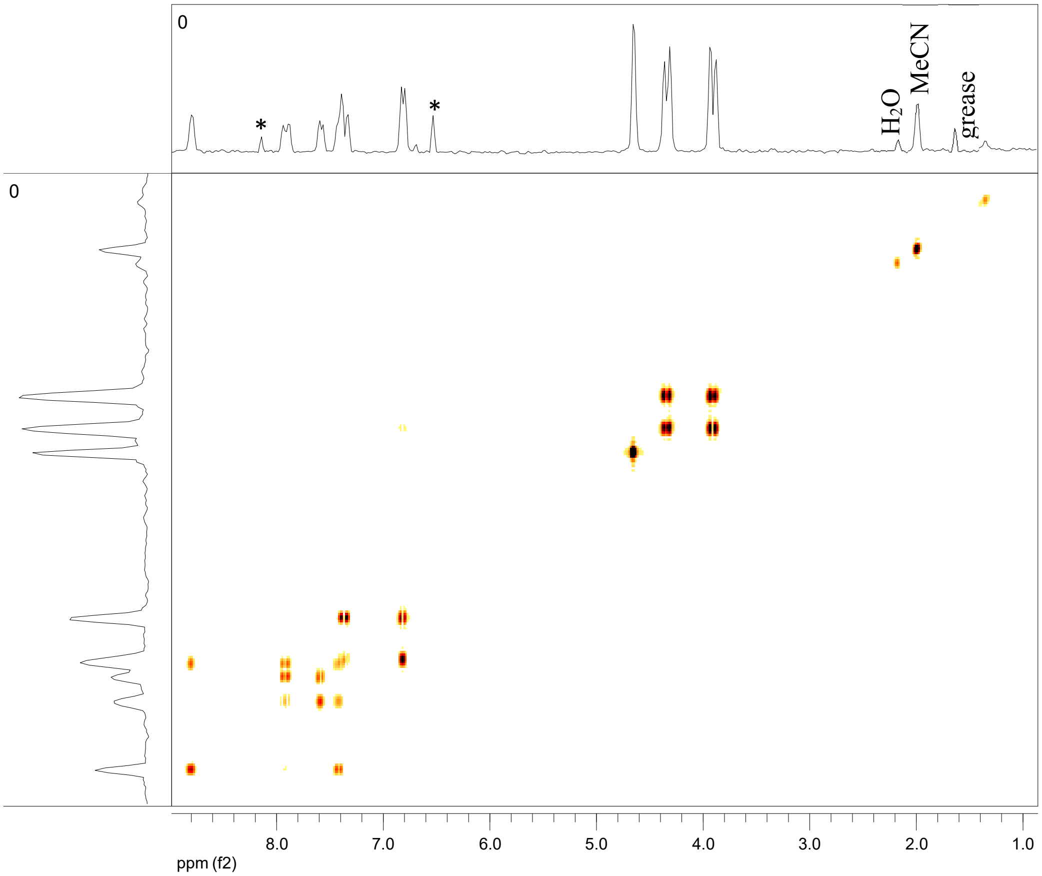


***Figure S11***. 300 MHz gCOSY NMR spectrum of [($^{Pic}$N4)Cu$^{I}$](OTf) in $CD_3CN$. Asterisk peaks are not present in the representative $^1$H spectrum.

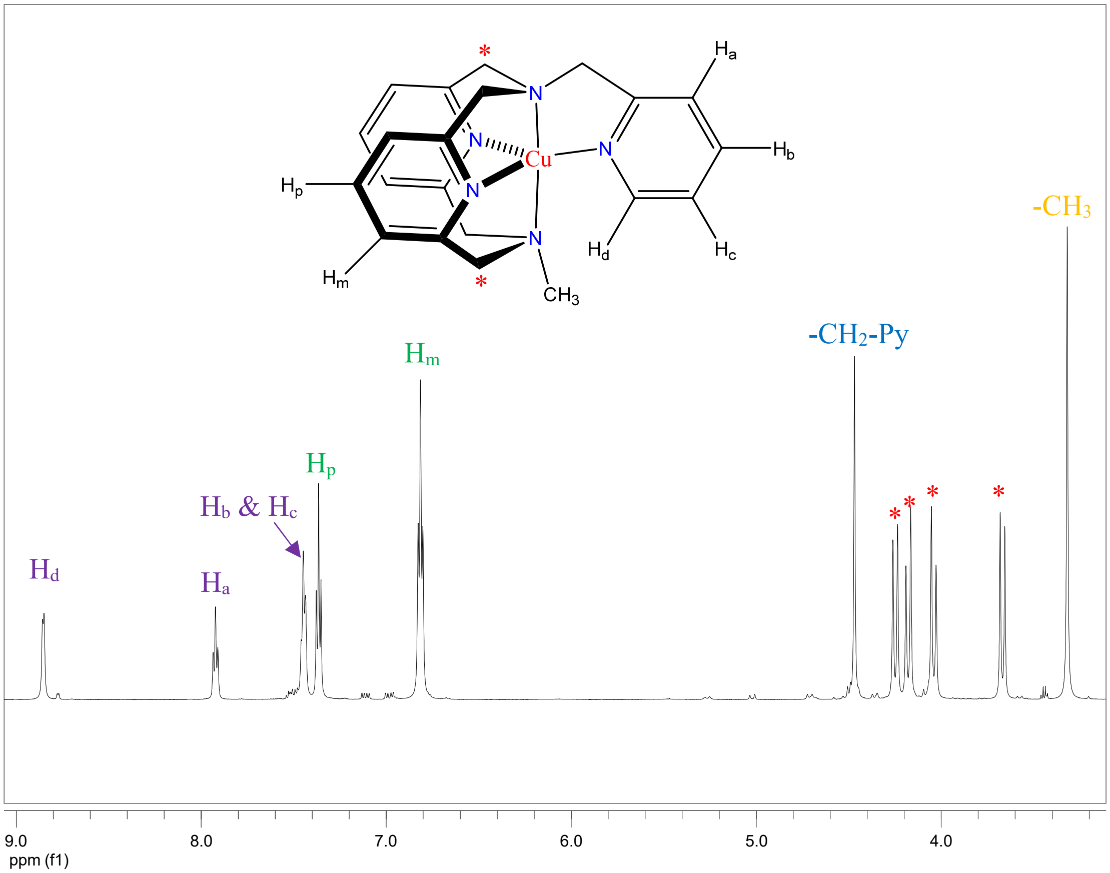


***Figure S12***. 600 MHz $^{1}$H-NMR spectrum of [($^{PicMe}$N4)Cu$^{I}$](OTf) in $CD_3CN$ with assignments. * corresponds with $CH_2$ on the N4 backbone.

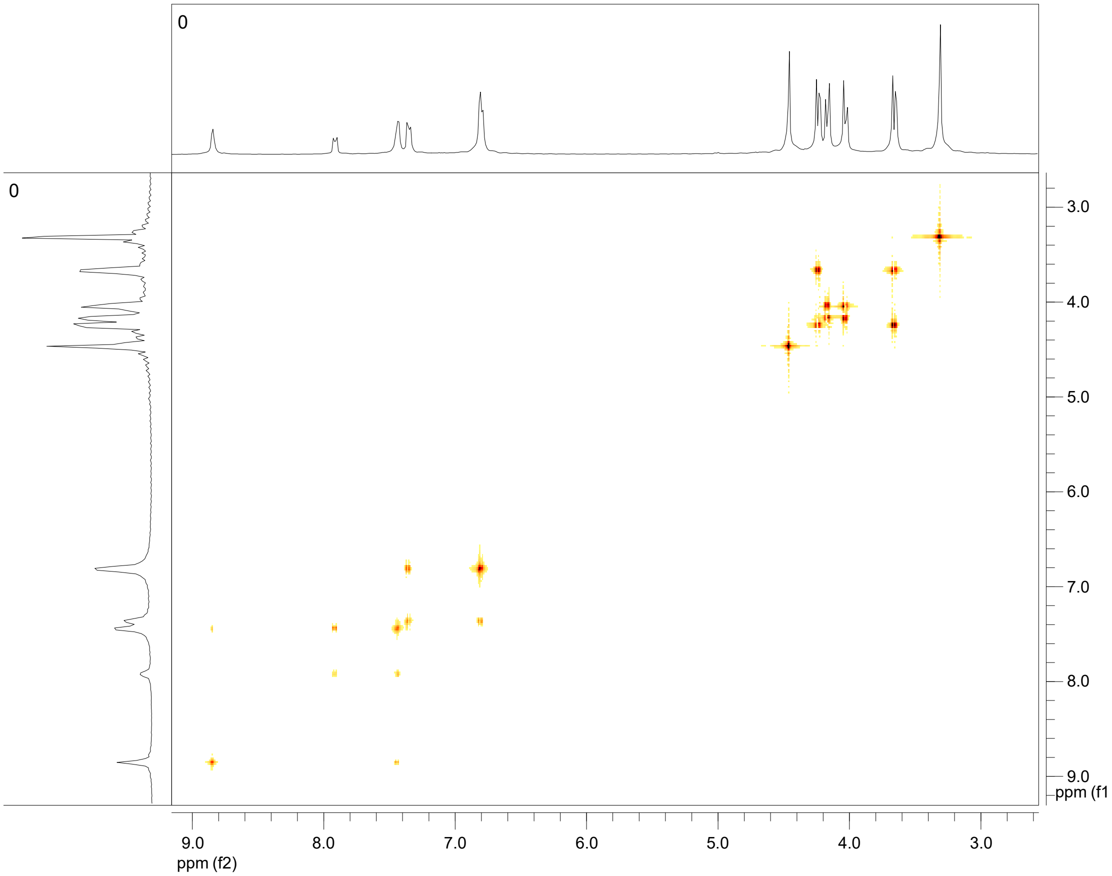


***Figure S13.*** 600 MHz gCOSY NMR spectrum of [($^{PicMe}$N4)Cu$^{I}$](OTf) in $CD_3CN$.

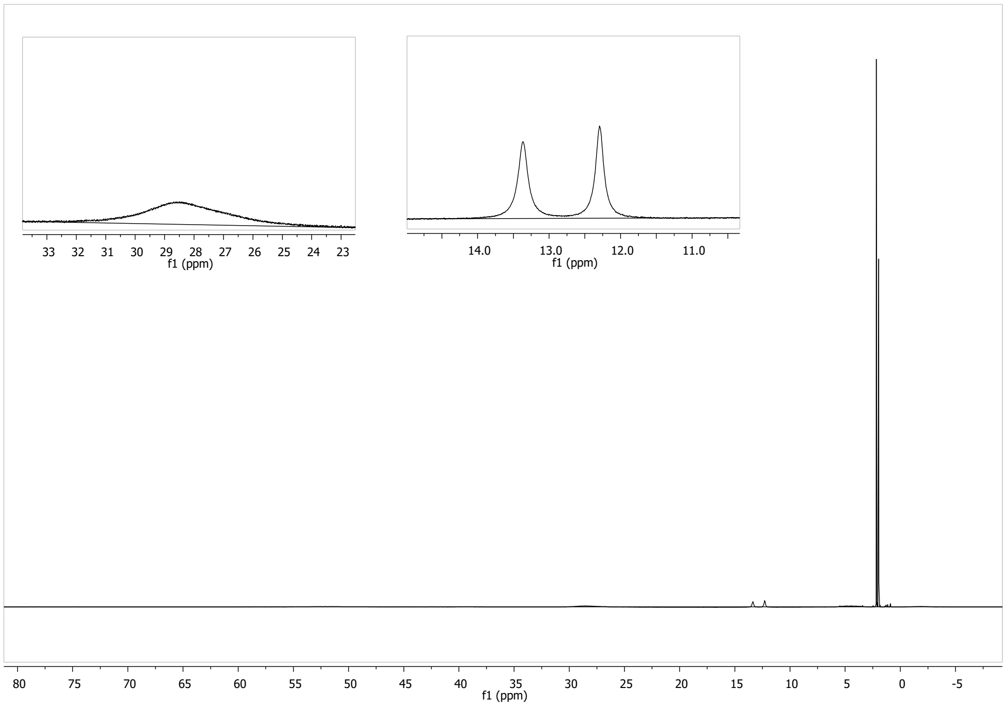


***Figure S14***. 600 MHz $^{1}$H-NMR spectrum of $[(^{Pic}N4)Cu^{II}](OTf)_2$ in $CD_3CN$.

## III. Cyclic voltammetry studies

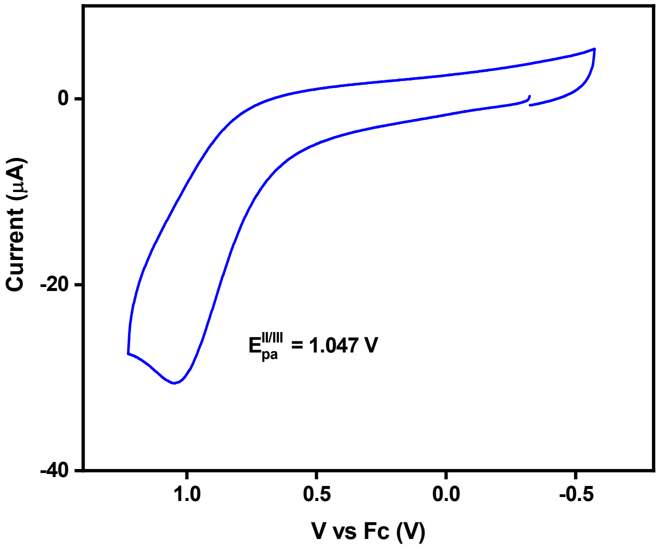


***Figure S15***. CV of **1**$^{2+}$ in 0.2M $Bu_4NClO_4/CH_3CN$, 100 mV/s scan rate, 3 sweeps.

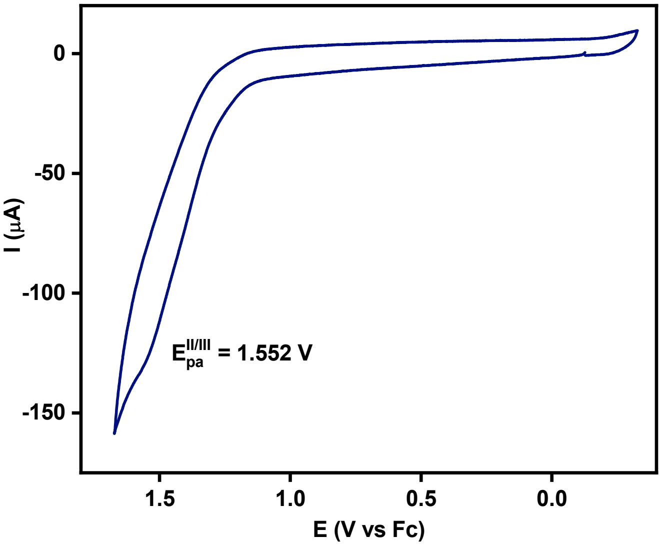


***Figure S16***. CV of **3**$^{2+}$ in 0.2M $Bu_4NClO_4/CH_3CN$, 100 mV/s scan rate, 3 sweeps.

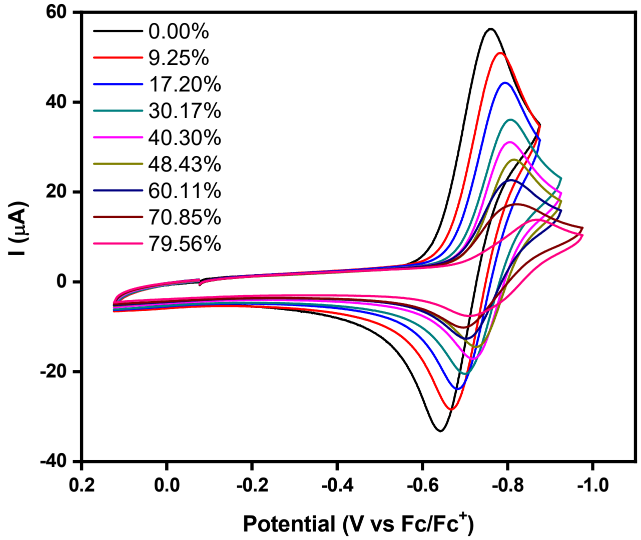


***Figure S17***. CV of **1**$^{2+}$ in 0.2M $Bu_4NClO_4/CH_3CN$ with increasing percentage of $H_2O$ (DI, $N_2$ purged). ($E_{1/2}{}^{II/I}$ = -0.752 V vs $Fc/Fc^+$, 100 mV/s scan rate, 3 sweeps, Ref: 0.01M $Ag/AgNO_3$).*

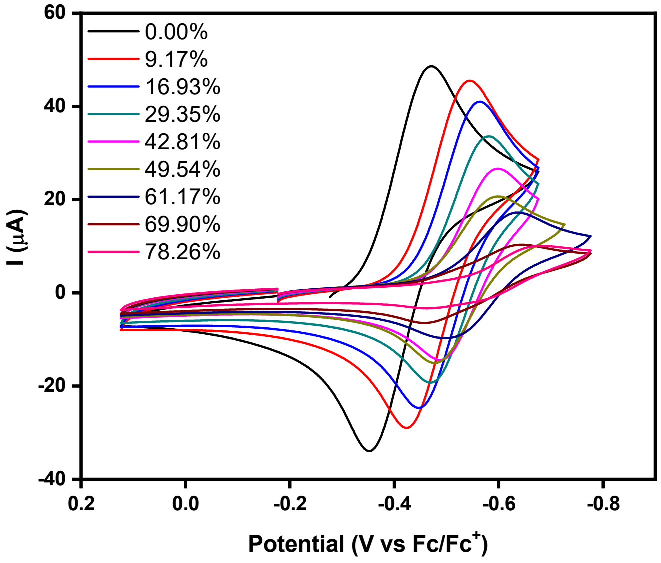


***Figure S18***. CV of **3**$^{2+}$ in 0.2M $Bu_4NClO_4/CH_3CN$ with increasing percentage of $H_2O$ (DI, $N_2$ purged). ($E_{1/2}{}^{II/I}$ = -0.412 V vs $Fc/Fc^+$, 100 mV/s scan rate, 3 sweeps, Ref: 0.01M $Ag/AgNO_3$).

**Note: decreasing concentration of the sample was due to water additions.

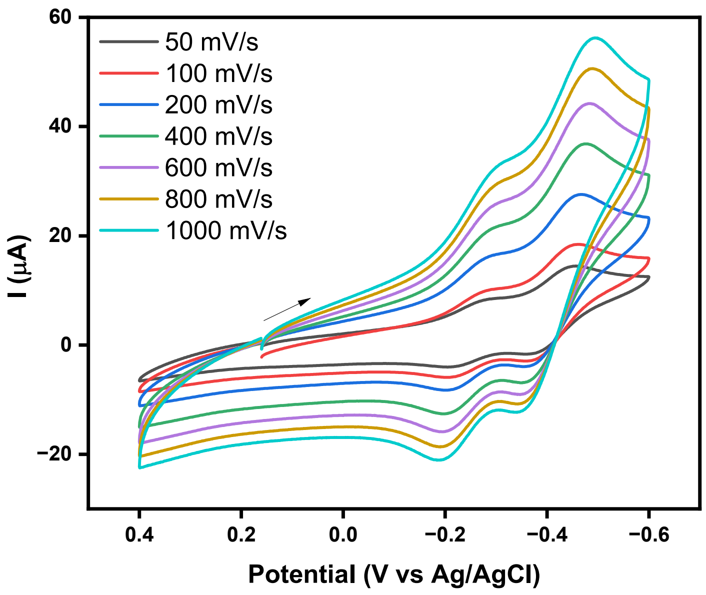


***Figure S19***. CV of **1**$^{2+}$ in 1:1 MeCN:0.1M NaOAc/$H_2O$ at variable scan rates (50-1000 mV/s).

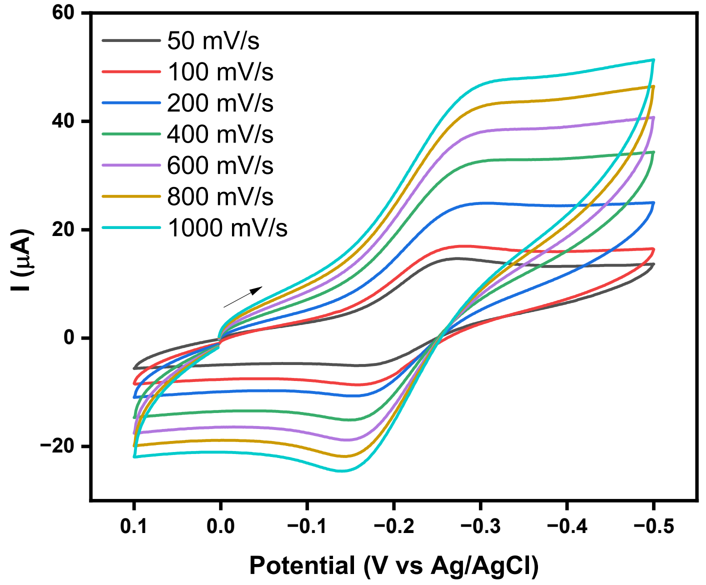


***Figure S20***. CV of **3**$^{2+}$ in 1:1 MeCN:0.1M NaOAc/$H_2O$ at variable scan rates (50-1000 mV/s).

## IV. UV-vis studies

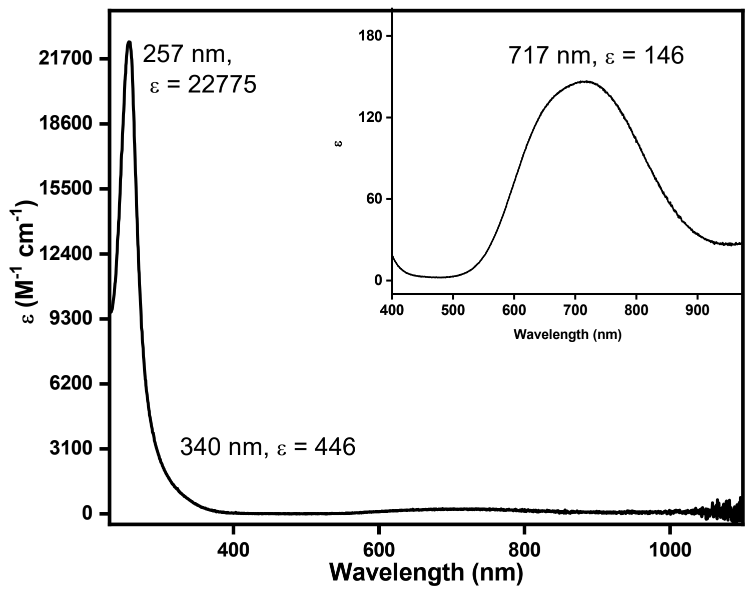


***Figure S21***. UV-vis spectrum of $\mathbf{1}^{2+}$ in MeCN at room temp.

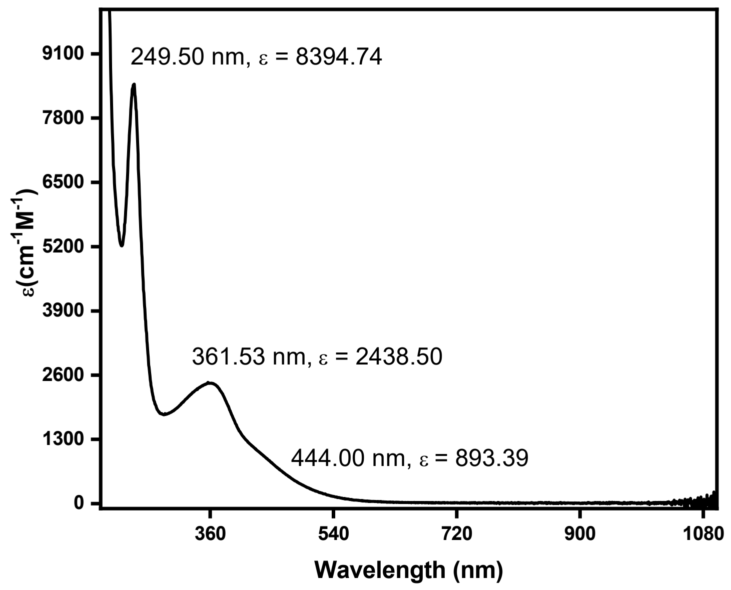


***Figure S22***. UV-vis spectrum of $\mathbf{2}^{+}$ in MeCN at room temp.

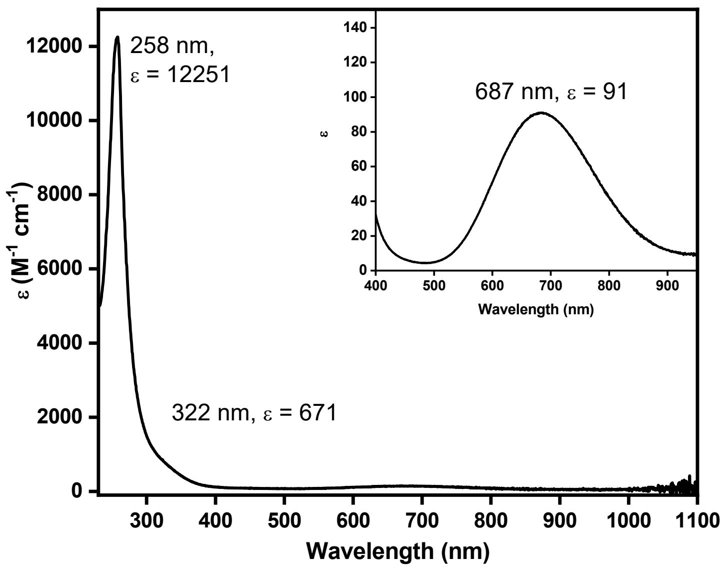


***Figure S23***. UV-vis spectrum of **3**$^{2+}$ in MeCN at room temp.

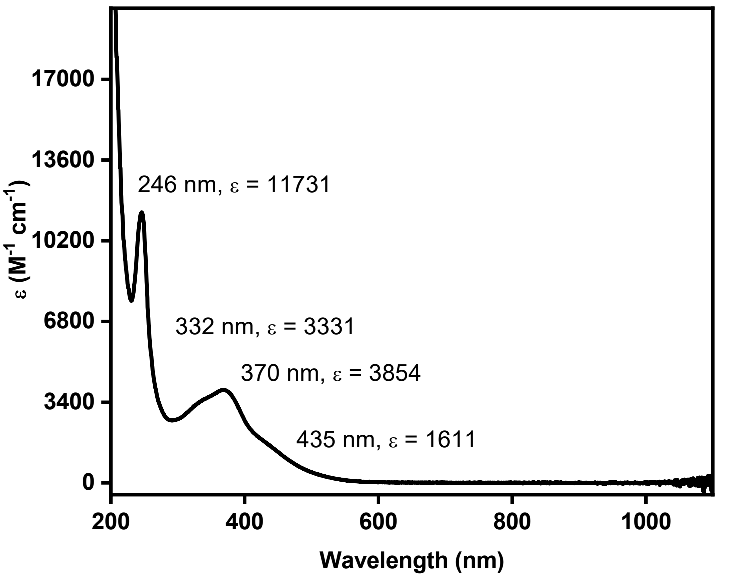


***Figure S24***. UV-vis spectrum of **4**$^{+}$ in MeCN at room temp.

## V. Acidity and stability constants determination

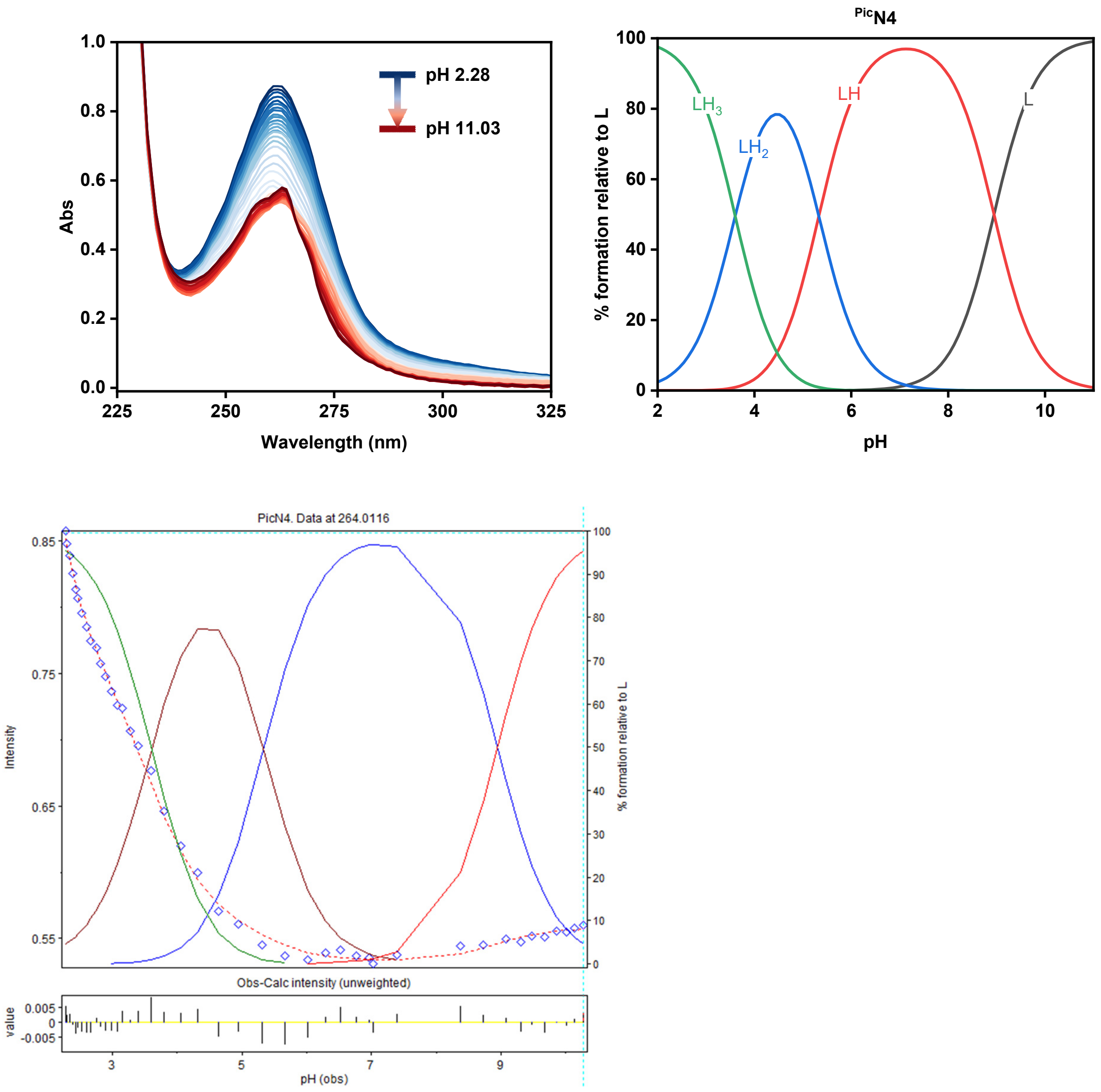


**Figure 24.** Variable pH (2.28-11.03) UV-Vis spectra of **$^{Pic}$N4**, its species distribution plot ([**$^{Pic}$N4**]$_{tot}$ = 60 µM), and HypSpec plot of pH vs absorbance at 264 nm with calculated fit.

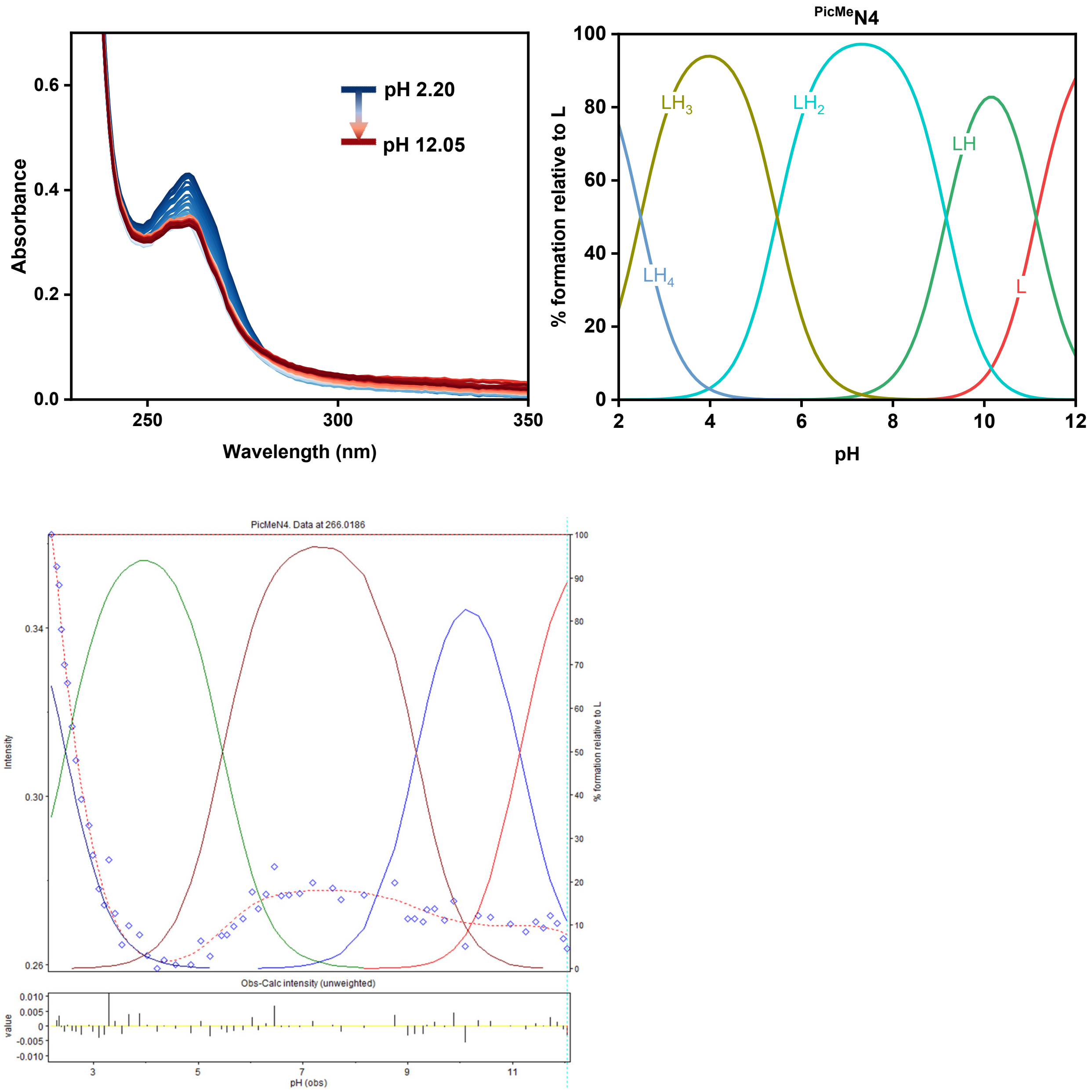


**Figure S25.** Variable pH (2.20-12.05) UV-Vis spectra of $^{PicMe}$**N4**, its species distribution plot ([$^{PicMe}$**N4**]$_{tot}$ = 60 µM), and HypSpec plot of pH vs absorbance at 266 nm with calculated fit.

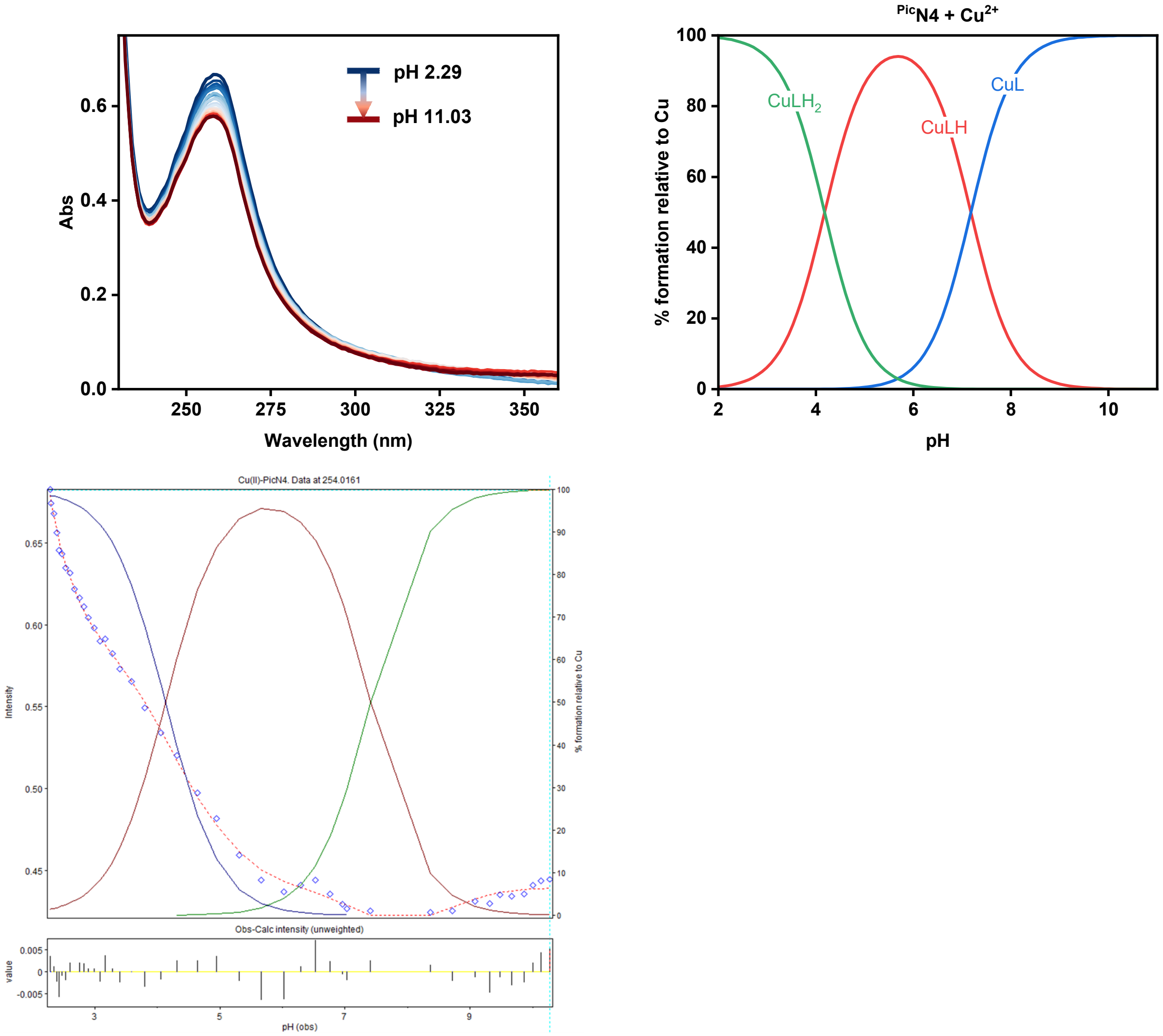


**Figure S26.** Variable pH (2.29-11.03) UV-Vis spectra of $^{Pic}$**N4** + $Cu^{2+}$, its species distribution plot ($[Cu^{2+}]_{tot}$ = [$^{Pic}$**N4**]$_{tot}$ = 60 µM), and HypSpec plot of pH vs. absorbance at 254 nm with calculated fit.

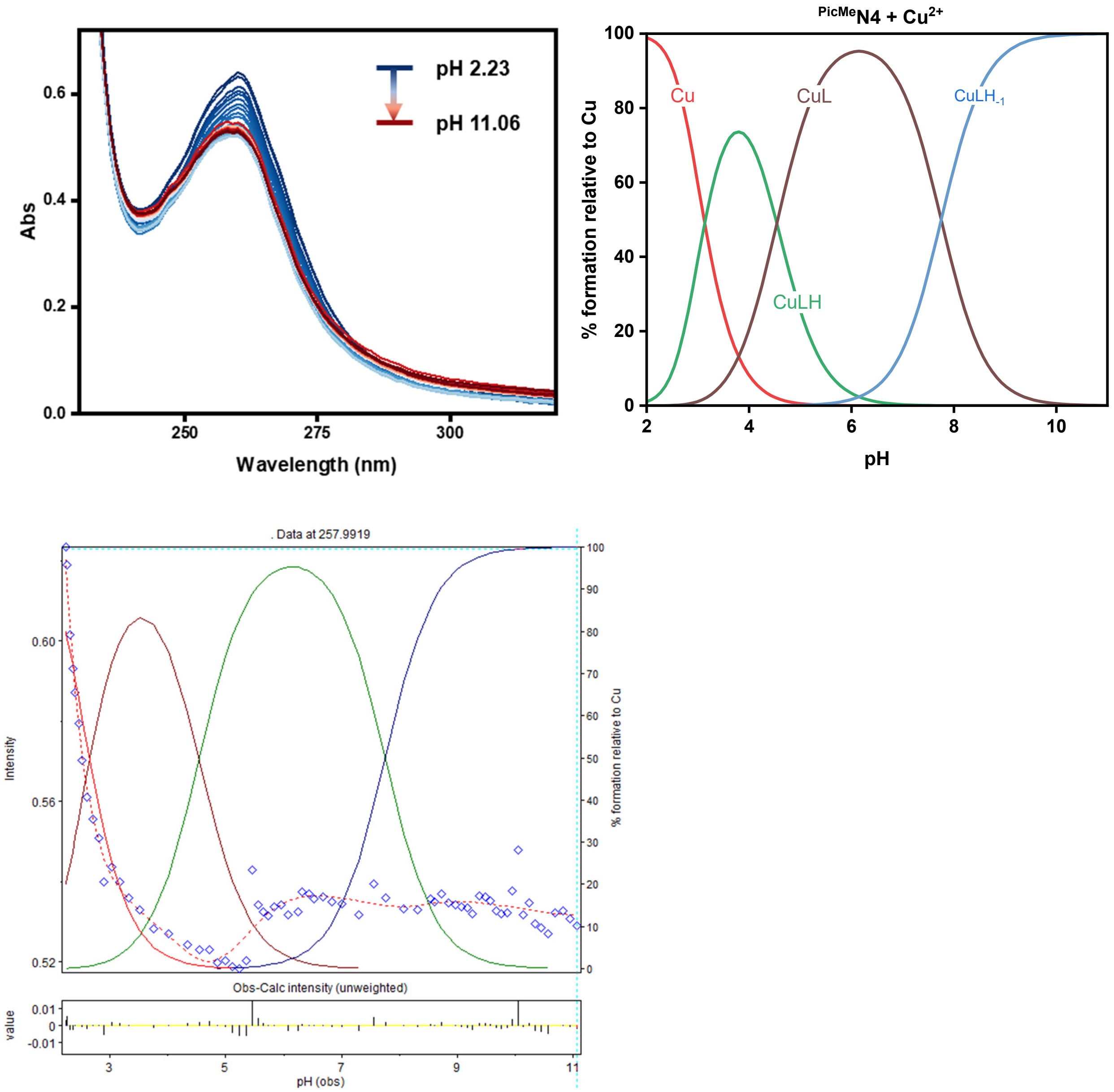


**Figure S27.** Variable pH (2.23-11.06) UV-Vis spectra of $^{PicMe}$**N4** + $Cu^{2+}$, its species distribution plot ($[Cu^{2+}]_{tot}$ = [$^{PicMe}$**N4**]$_{tot}$ = 60 µM), and HypSpec plot of pH vs. absorbance at 257 nm with calculated fit.

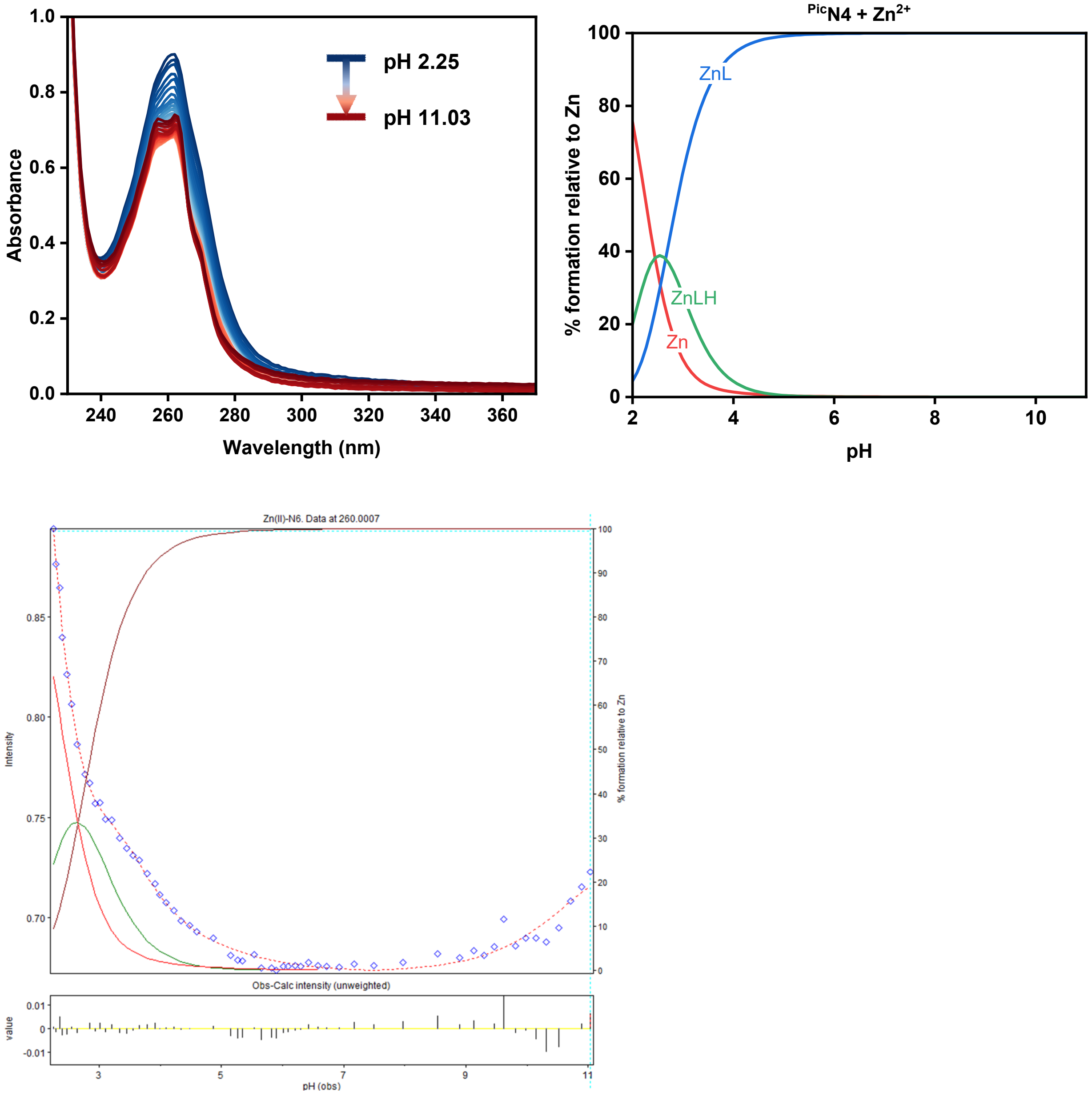


**Figure S28.** Variable pH (2.25-11.03) UV-Vis spectra of $^{Pic}$**N4 + Zn$^{2+}$** ([Zn$^{2+}$]$_{tot}$ = [$^{Pic}$**N4**]$_{tot}$ = 80 µM, its species distribution plot, and HypSpec plot of absorbance at 260 nm vs pH with calculated fit.

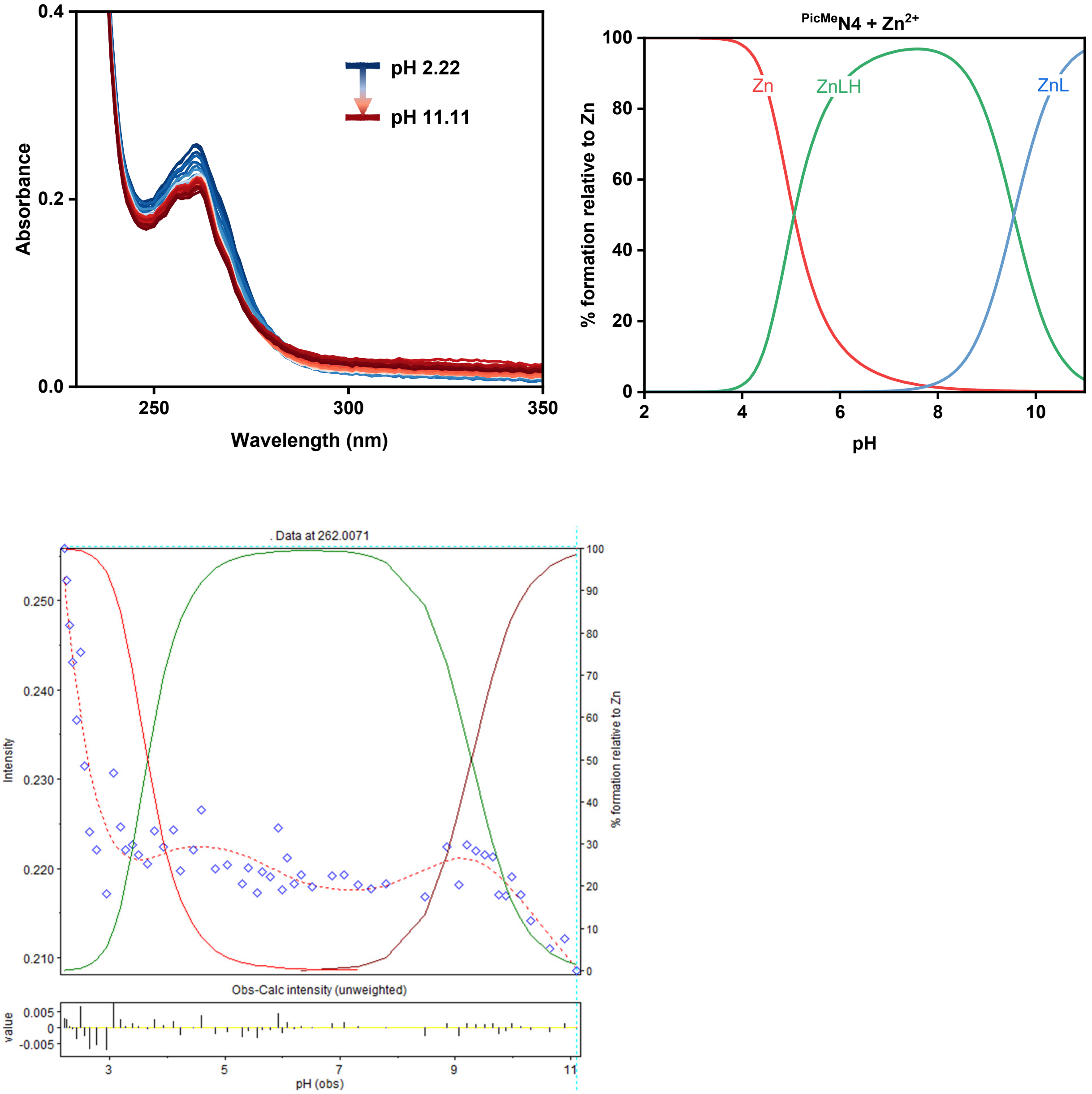


**Figure S29.** Variable pH (2.22-11.11) UV-Vis spectra of **$^{PicMe}$N4 + $Zn^{2+}$** ($[Zn^{2+}]_{tot}$ = $[^{PicMe}$**N4**$]_{tot}$ = 80 µM, its species distribution plot, and HypSpec plot of absorbance at 262 nm vs pH with calculated fit.

## VI. X-ray structure determination

### X-ray structure determination of [($^{Pic}$N4)Cu(II)]($PF_6$)$_2$

Table S4. Crystal data and structure refinement for [($^{Pic}$N4)$Cu^{II}$]($PF_6$)$_2$.

| | | |
|---|---|---|
| Identification code | l15613/lt/x8/100/AJW-Cu1 (Blue crystal) | |
| Empirical formula | $C_{28} H_{29} Cu F_{12} N_7 P_2$ | |
| Formula weight | 817.06 | |
| Temperature | 100(2) K | |
| Wavelength | 0.71073 Å | |
| Crystal system | Monoclinic | |
| Space group | P $2_1$ | |
| Unit cell dimensions | a = 8.4472(5) Å | α= 90°. |
| | b = 18.1995(9) Å | β= 90.242(4)°. |
| | c = 10.2897(6) Å | γ = 90°. |
| Volume | 1581.87(15) Å$^3$ | |
| Z | 2 | |
| Density (calculated) | 1.715 Mg/m$^3$ | |
| Absorption coefficient | 0.897 mm$^{-1}$ | |
| F(000) | 826 | |
| Crystal size | 0.251 x 0.194 x 0.128 mm$^3$ | |
| Theta range for data collection | 2.238 to 30.876°. | |
| Index ranges | -12≤h≤12, -26≤k≤26, -14≤l≤14 | |
| Reflections collected | 24563 | |
| Independent reflections | 9919 [R(int) = 0.0517] | |
| Completeness to theta = 25.242° | 99.9 % | |
| Absorption correction | Semi-empirical from equivalents | |
| Max. and min. transmission | 0.8622 and 0.7701 | |
| Refinement method | Full-matrix least-squares on F$^2$ | |
| Data / restraints / parameters | 9919 / 127 / 452 | |
| Goodness-of-fit on F$^2$ | 1.026 | |
| Final R indices [I>2sigma(I)] | R1 = 0.0499, wR2 = 0.1031 | |
| R indices (all data) | R1 = 0.0718, wR2 = 0.1148 | |
| Absolute structure parameter | 0.009(7) | |
| Extinction coefficient | n/a | |
| Largest diff. peak and hole | 0.637 and -0.615 e.Å$^{-3}$ | |

Table S5. Bond lengths [Å] for [($^{Pic}$N4)Cu$^{II}$](PF$_6$)$_2$.

| | |
|---|---|
| Cu(1)-N(3) | 2.003(4) |
| Cu(1)-N(6) | 2.017(4) |
| Cu(1)-N(2) | 2.028(4) |
| Cu(1)-N(1) | 2.056(4) |
| Cu(1)-N(4) | 2.276(4) |
| Cu(1)-N(5) | 2.348(4) |
| | |
| N(3)-Cu(1)-N(6) | 92.94(15) |
| N(3)-Cu(1)-N(2) | 156.22(16) |
| N(6)-Cu(1)-N(2) | 96.07(15) |
| N(3)-Cu(1)-N(1) | 97.29(15) |
| N(6)-Cu(1)-N(1) | 153.02(16) |
| N(2)-Cu(1)-N(1) | 84.43(15) |
| N(3)-Cu(1)-N(4) | 78.26(15) |
| N(6)-Cu(1)-N(4) | 127.22(15) |
| N(2)-Cu(1)-N(4) | 78.76(15) |
| N(1)-Cu(1)-N(4) | 79.45(15) |
| N(3)-Cu(1)-N(5) | 125.10(15) |
| N(6)-Cu(1)-N(5) | 76.93(14) |
| N(2)-Cu(1)-N(5) | 78.47(15) |
| N(1)-Cu(1)-N(5) | 76.75(14) |
| N(4)-Cu(1)-N(5) | 148.38(14) |

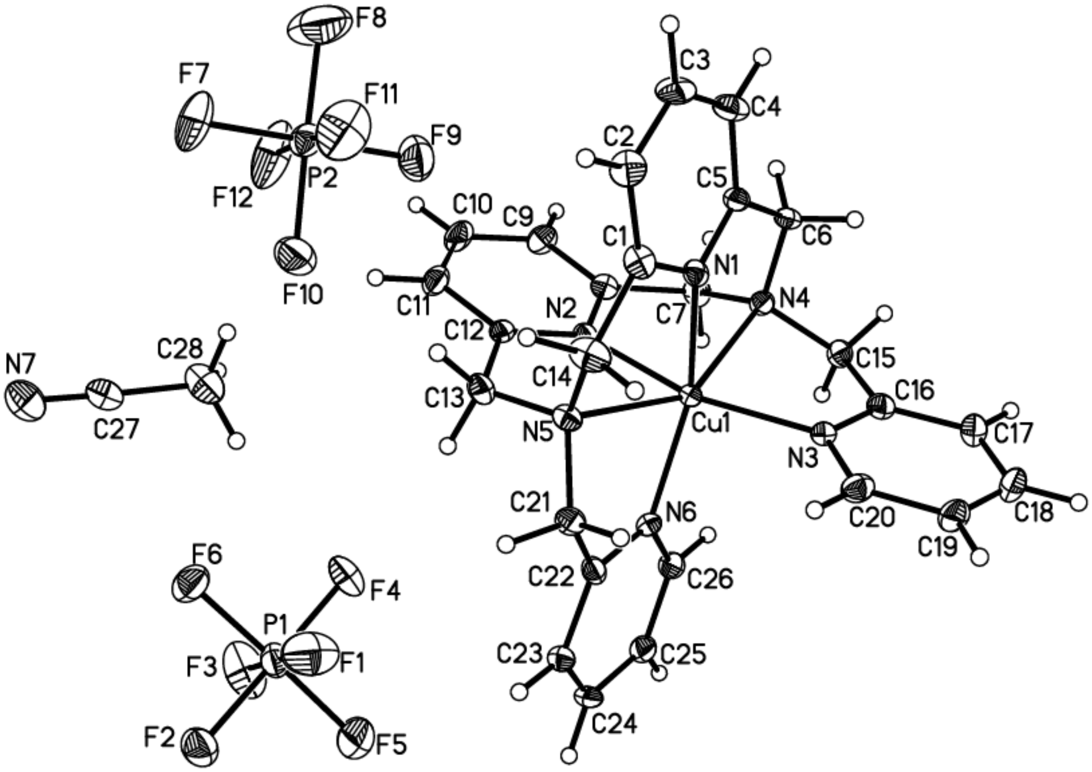


***Figure S30***. Projection view with 50% probability.

## X-ray structure determination of [($^{Pic}$N4)Cu(I)]($PF_6$)$_2$.

Table S6. Crystal data and structure refinement for [($^{Pic}$N4)Cu$^{I}$]($PF_6$)$_2$.

| | | |
|---|---|---|
| Identification code | l15513/lt/x8/100K/AJW-Cu1 | |
| Empirical formula | $C_{26}$ $H_{26}$ Cu $F_6$ $N_6$ P | |
| Formula weight | 631.04 | |
| Temperature | 100(2) K | |
| Wavelength | 0.71073 Å | |
| Crystal system | Monoclinic | |
| Space group | P $2_1$/n | |
| Unit cell dimensions | a = 10.3737(8) Å | α= 90°. |
| | b = 11.4183(8) Å | β= 101.859(4)°. |
| | c = 21.8851(15) Å | γ = 90°. |
| Volume | 2537.0(3) Å$^3$ | |
| Z | 4 | |
| Density (calculated) | 1.652 Mg/m$^3$ | |
| Absorption coefficient | 0.998 mm$^{-1}$ | |
| F(000) | 1288 | |
| Crystal size | 0.269 x 0.221 x 0.202 mm$^3$ | |
| Theta range for data collection | 1.902 to 28.407°. | |
| Index ranges | -13≤h≤13, -15≤k≤15, -29≤l≤29 | |
| Reflections collected | 40019 | |
| Independent reflections | 6364 [R(int) = 0.0430] | |
| Completeness to theta = 25.242° | 100.0 % | |
| Absorption correction | Semi-empirical from equivalents | |
| Max. and min. transmission | 0.8621 and 0.7904 | |
| Refinement method | Full-matrix least-squares on F$^2$ | |
| Data / restraints / parameters | 6364 / 0 / 361 | |
| Goodness-of-fit on F$^2$ | 1.032 | |
| Final R indices [I>2sigma(I)] | R1 = 0.0290, wR2 = 0.0656 | |
| R indices (all data) | R1 = 0.0401, wR2 = 0.0700 | |
| Extinction coefficient | n/a | |
| Largest diff. peak and hole | 0.421 and -0.292 e.Å$^{-3}$ | |

Table S7. Bond lengths [Å] for [($^{Pic}$N4)Cu$^{I}$]($PF_6$)$_2$.

| | |
|---|---|
| Cu(1)-N(3) | 1.9640(13) |
| Cu(1)-N(2) | 2.0817(13) |
| Cu(1)-N(1) | 2.1341(13) |
| Cu(1)-N(5) | 2.3456(14) |
| Cu(1)-N(4) | 2.3983(14) |

| | |
|---|---|
| N(3)-Cu(1)-N(2) | 139.16(6) |
| N(3)-Cu(1)-N(1) | 123.74(5) |
| N(2)-Cu(1)-N(1) | 81.36(5) |
| N(3)-Cu(1)-N(5) | 133.44(5) |
| N(2)-Cu(1)-N(5) | 78.96(5) |
| N(1)-Cu(1)-N(5) | 77.79(5) |
| N(3)-Cu(1)-N(4) | 79.12(5) |
| N(2)-Cu(1)-N(4) | 76.30(5) |
| N(1)-Cu(1)-N(4) | 76.87(5) |
| N(5)-Cu(1)-N(4) | 146.78(5) |

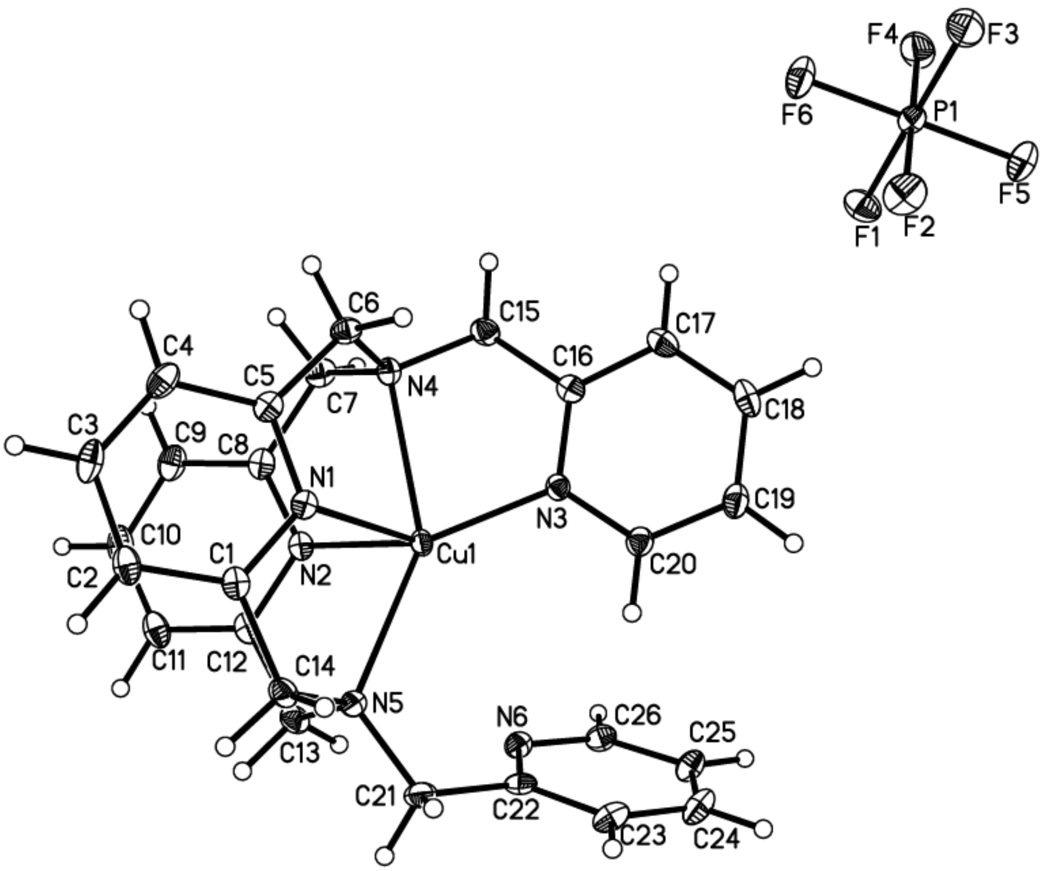


***Figure S31***.Projection view with 50% probability ellipsoids:

## X-ray structure determination of $[(^{PicMe}N4)Cu^{II}](OTf)_2$.

Table S8. Crystal data and structure refinement for $[(^{PicMe}N4)Cu^{II}](OTf)_2$..

| | | |
|---|---|---|
| Identification code | l7215/lt/smart/AJW-N5CuIIOTf | |
| Empirical formula | C71 H66 B2 Cu N6 | |
| Formula weight | 1088.45 | |
| Temperature | 100(2) K | |
| Wavelength | 0.71073 Å | |
| Crystal system | Monoclinic | |
| Space group | Pn | |
| Unit cell dimensions | a = 12.4202(6) Å | [illegible]= 90°. |
| | b = 18.1170(8) Å | [illegible]= 91.082(2)°. |
| | c = 12.6237(5) Å | [illegible] = 90°. |
| Volume | 2840.0(2) $Å^3$ | |
| Z | 2 | |
| Density (calculated) | 1.273 $Mg/m^3$ | |
| Absorption coefficient | 0.434 $mm^{-1}$ | |
| F(000) | 1146 | |
| Crystal size | 0.213 x 0.182 x 0.088 $mm^3$ | |
| Theta range for data collection | 1.966 to 27.109°. | |
| Index ranges | -15≤h≤15, -23≤k≤23, -16≤l≤16 | |
| Reflections collected | 69835 | |
| Independent reflections | 12487 [R(int) = 0.0600] | |
| Completeness to theta = 25.242° | 99.9 % | |
| Absorption correction | Semi-empirical from equivalents | |
| Max. and min. transmission | 0.7455 and 0.6941 | |
| Refinement method | Full-matrix least-squares on $F^2$ | |
| Data / restraints / parameters | 12487 / 118 / 716 | |
| Goodness-of-fit on $F^2$ | 1.030 | |
| Final R indices [I>2sigma(I)] | R1 = 0.0693, wR2 = 0.1778 | |
| R indices (all data) | R1 = 0.0940, wR2 = 0.2007 | |
| Absolute structure parameter | 0.058(5) | |
| Extinction coefficient | n/a | |
| Largest diff. peak and hole | 0.782 and -0.606 e.$Å^{-3}$ | |

Table S9. Bond lengths [Å] and angles [°] for $[(^{PicMe}N4)Cu^{II}](OTf)_2$.

| | | | |
|---|---|---|---|
| Cu(1)-N(1) | 1.944(9) | N(2)-Cu(1)-N(6) | 168.4(4) |
| Cu(1)-N(3) | 1.967(8) | N(1)-Cu(1)-N(4) | 82.0(5) |
| Cu(1)-N(5) | 2.165(8) | N(3)-Cu(1)-N(4) | 115.6(4) |
| Cu(1)-N(2) | 2.173(7) | N(5)-Cu(1)-N(4) | 152.1(3) |
| Cu(1)-N(6) | 2.219(9) | N(2)-Cu(1)-N(4) | 77.5(3) |
| Cu(1)-N(4) | 2.258(7) | N(6)-Cu(1)-N(4) | 91.7(4) |
| Cu(1')-N(1') | 1.966(12) | N(1')-Cu(1')-N(3) | 173.6(9) |
| Cu(1')-N(3) | 2.015(9) | N(1')-Cu(1')-N(2) | 82.5(10) |
| Cu(1')-N(2) | 2.091(10) | N(3)-Cu(1')-N(2) | 101.4(4) |
| Cu(1')-N(5) | 2.096(10) | N(1')-Cu(1')-N(5) | 92.5(10) |
| Cu(1')-N(4) | 2.285(8) | N(3)-Cu(1')-N(5) | 83.1(4) |
| Cu(1')-N(6) | 2.305(11) | N(2)-Cu(1')-N(5) | 81.8(4) |
| | | N(1')-Cu(1')-N(4) | 73.2(8) |
| N(1)-Cu(1)-N(3) | 162.5(5) | N(3)-Cu(1')-N(4) | 112.5(5) |
| N(1)-Cu(1)-N(5) | 81.3(6) | N(2)-Cu(1')-N(4) | 78.6(3) |
| N(3)-Cu(1)-N(5) | 82.4(4) | N(5)-Cu(1')-N(4) | 156.9(5) |
| N(1)-Cu(1)-N(2) | 82.9(6) | N(1')-Cu(1')-N(6) | 92.3(11) |
| N(3)-Cu(1)-N(2) | 100.1(4) | N(3)-Cu(1')-N(6) | 84.9(5) |
| N(5)-Cu(1)-N(2) | 78.4(3) | N(2)-Cu(1')-N(6) | 167.3(4) |
| N(1)-Cu(1)-N(6) | 91.3(6) | N(5)-Cu(1')-N(6) | 110.1(4) |
| N(3)-Cu(1)-N(6) | 88.4(4) | N(4)-Cu(1')-N(6) | 88.9(4) |
| N(5)-Cu(1)-N(6) | 110.8(4) | | |

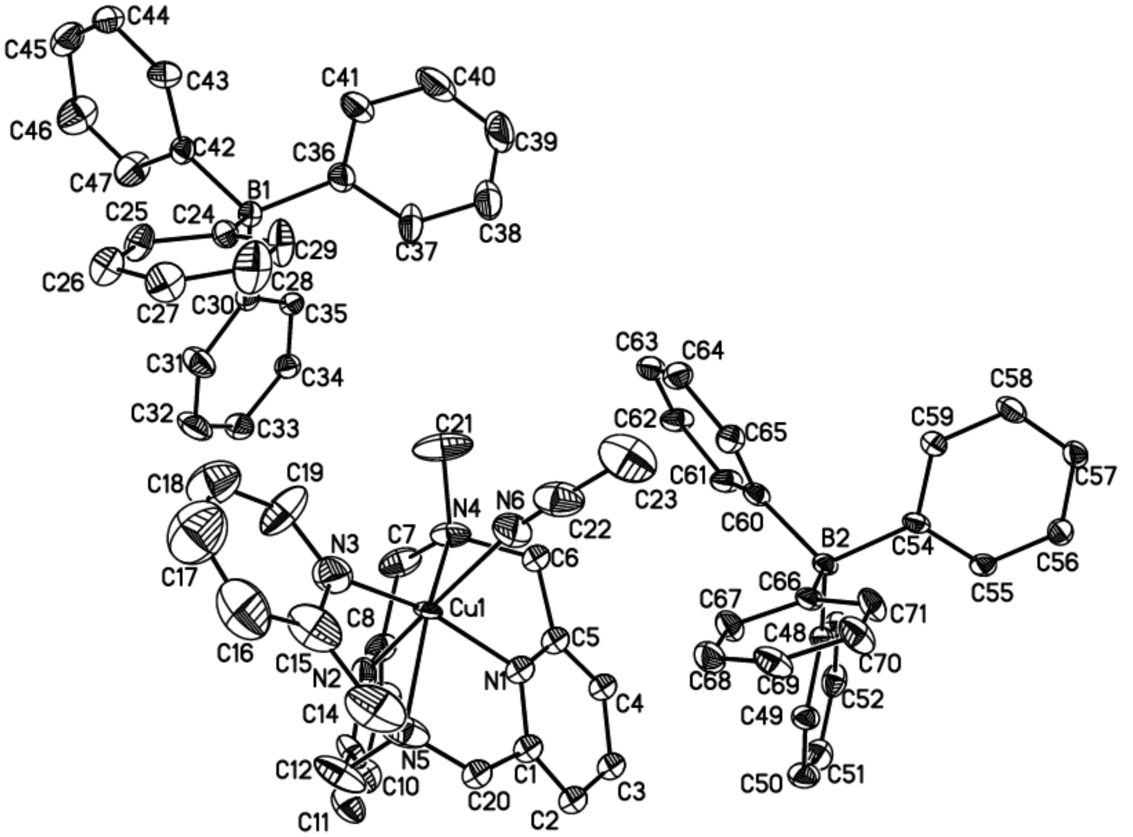


***Figure S32***.Projection view with 30% probability ellipsoids: H atoms and disorder components omitted for clarity.

## X-ray structure determination of $[(^{PicMe}N4)Cu^{I}](ClO_4)$.

Table S10. Crystal data and structure refinement for $[(^{PicMe}N4)Cu^{I}](ClO_4)$.

| | | |
|---|---|---|
| Identification code | l16513/lt/x8/AJW-Cu1 | |
| Empirical formula | $C_{21}$ $H_{23}$ Cl Cu $N_5$ $O_4$ | |
| Formula weight | 508.43 | |
| Temperature | 100(2) K | |
| Wavelength | 0.71073 Å | |
| Crystal system | Triclinic | |
| Space group | P $\bar{1}$ | |
| Unit cell dimensions | a = 8.0078(4) Å | [illegible]= 101.005(3)°. |
| | b = 10.5316(6) Å | [illegible]= 92.977(3)°. |
| | c = 12.7193(7) Å | [illegible] = 93.097(3)°. |
| Volume | 1049.29(10) $Å^3$ | |
| Z | 2 | |
| Density (calculated) | 1.609 $Mg/m^3$ | |
| Absorption coefficient | 1.209 $mm^{-1}$ | |
| F(000) | 524 | |
| Crystal size | 0.266 x 0.205 x 0.133 $mm^3$ | |
| Theta range for data collection | 1.974 to 28.766°. | |
| Index ranges | -10≤h≤10, -14≤k≤14, -17≤l≤17 | |
| Reflections collected | 25672 | |
| Independent reflections | 5450 [R(int) = 0.0321] | |
| Completeness to theta = 25.242° | 99.9 % | |
| Absorption correction | Semi-empirical from equivalents | |
| Max. and min. transmission | 0.8621 and 0.7802 | |
| Refinement method | Full-matrix least-squares on $F^2$ | |
| Data / restraints / parameters | 5450 / 74 / 305 | |
| Goodness-of-fit on $F^2$ | 1.028 | |
| Final R indices [I>2sigma(I)] | R1 = 0.0318, wR2 = 0.0765 | |
| R indices (all data) | R1 = 0.0392, wR2 = 0.0801 | |
| Extinction coefficient | n/a | |
| Largest diff. peak and hole | 0.965 and -0.292 $e.Å^{-3}$ | |

Table S11. Bond lengths [Å] and angles [°] for [($^{PicMe}$N4)$Cu^{I}$]($ClO_4$).

| | |
|---|---|
| Cu(1)-N(3) | 1.9461(14) |
| Cu(1)-N(2) | 2.0768(15) |
| Cu(1)-N(1) | 2.1286(14) |
| Cu(1)-N(5) | 2.262(2) |
| Cu(1)-N(5') | 2.37(4) |
| Cu(1)-N(4) | 2.3957(15) |
| | |
| N(3)-Cu(1)-N(2) | 140.61(6) |
| N(3)-Cu(1)-N(1) | 121.91(6) |
| N(2)-Cu(1)-N(1) | 83.06(6) |
| N(3)-Cu(1)-N(5) | 131.36(6) |
| N(2)-Cu(1)-N(5) | 79.44(6) |
| N(1)-Cu(1)-N(5) | 78.92(6) |
| N(3)-Cu(1)-N(5') | 128.4(5) |
| N(2)-Cu(1)-N(5') | 81.9(4) |
| N(1)-Cu(1)-N(5') | 80.7(4) |
| N(3)-Cu(1)-N(4) | 80.15(5) |
| N(2)-Cu(1)-N(4) | 76.33(5) |
| N(1)-Cu(1)-N(4) | 77.45(5) |
| N(5)-Cu(1)-N(4) | 147.84(6) |
| N(5')-Cu(1)-N(4) | 150.7(5) |

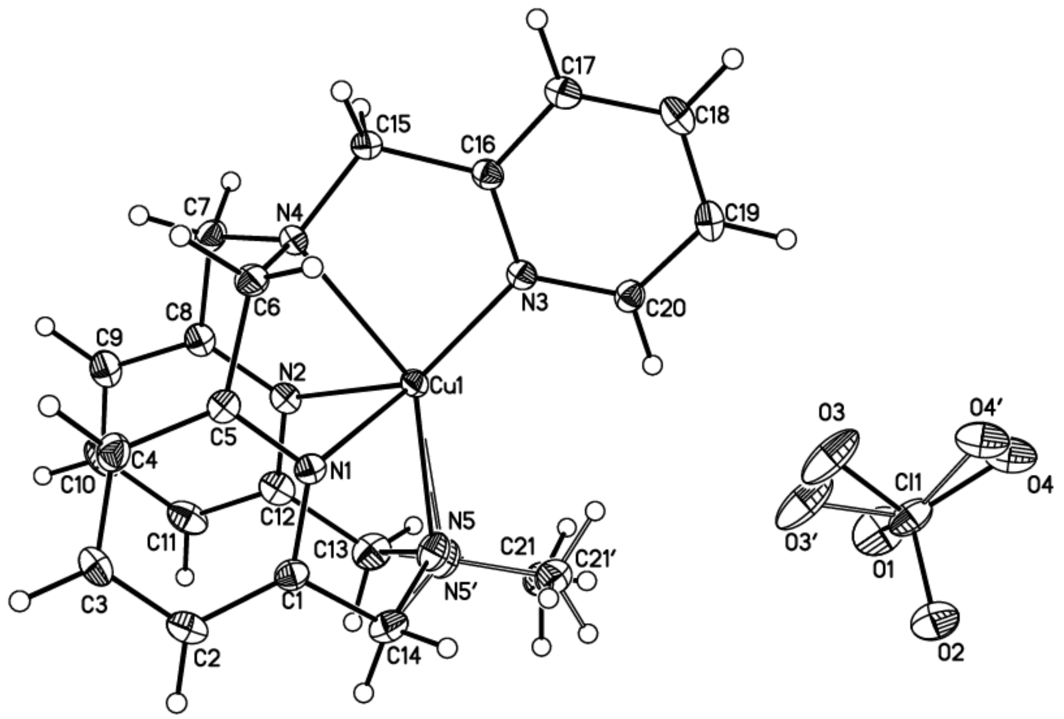


***Figure S33.*** Projection view with 50% probability ellipsoids:

## VI. References


1. Bottino, F.; Di Grazia, M.; Finocchiaro, P.; Fronczek, F. R.; Mamo, A.; Pappalardo, S., Reaction of Tosylamide Monosodium Salt with Bis(halomethyl) Compounds: an Easy Entry to Symmetrical N-tosylazamacrocycles. *Journal of Organic Chemistry* **1988,** *53* (15), 3521-9.
2. Irangu, J.; Ferguson, M. J.; Jordan, R. B., Reaction of copper(II) with ferrocene and 1,1 '-dimethylferrocene in aqueous acetonitrile: The copper(II/I) self-exchange rate. *Inorg. Chem.* **2005,** *44* (5), 1619-1625.
3. Evans, D. F., Determination of the Paramagnetic Susceptibility of Substances in Solution By NMR. *J. Chem. Soc.* **1959**, 2003-2005.
4. De Buysser, K.; Herman, G. G.; Bruneel, E.; Hoste, S.; Van Driessche, I., Determination of the Number of Unpaired Electrons in Metal-Complexes. A Comparison Between the Evans' Method and Susceptometer Results. *Chem. Phys.* **2005,** *315* (3), 286-292.
5. Bain, G. A.; Berry, J. F., Diamagnetic Corrections and Pascal's Constants. *J. Chem. Educ.* **2008,** *85* (4), 532-536.
6. Krause, L.; Herbst-Irmer, R.; Sheldrick, G. M.; Stalke, D., Comparison of silver and molybdenum microfocus X-ray sources for single-crystal structure determination. *J. Appl. Crystallogr.* **2015,** *48*, 3-10.
7. Sheldrick, G., SHELXT - Integrated space-group and crystal-structure determination. *Acta Crystallographica Section A* **2015,** *71* (1), 3-8.
8. Alderighi, L.; Gans, P.; Ienco, A.; Peters, D.; Sabatini, A.; Vacca, A., Hyperquad simulation and speciation (HySS): a utility program for the investigation of equilibria involving soluble and partially soluble species. *Coord. Chem. Rev.* **1999,** *184* (1), 311-318.
9. Bagchi, P.; Morgan, M. T.; Bacsa, J.; Fahrni, C. J., Robust Affinity Standards for Cu(I) Biochemistry. *J. Am. Chem. Soc.* **2013,** *135* (49), 18549-18559.
10. Halfen, J. A.; Tolman, W. B.; Weighardt, K., C2-Symmetric 1,4-Diisopropyl-7-R-1,4,7-Triazacyclononanes. In *Inorg. Synth.*, John Wiley & Sons, Inc.: 2007; pp 75-81.
